# Synthesis of Azo Disperse Dyes with High Absorption for Efficient Polyethylene Terephthalate Dyeing Performances in Supercritical Carbon Dioxide

**DOI:** 10.3390/polym14153020

**Published:** 2022-07-26

**Authors:** Yu-Wen Cheng, Jean-Sebastien Benas, Fang-Cheng Liang, Shang-Ming Lin, Ting-Wang Sun, Fu-Chieh Liu, Yang-Yen Yu, Chi-Ching Kuo

**Affiliations:** 1 Institute of Organic and Polymeric Materials, Research and Development Center of Smart Textile Technology, National Taipei University of Technology, No. 1, Sec. 3, Chung-Hsiao East Road, Taipei 10608, Taiwan; weven007@yahoo.com.tw (Y.-W.C.); benas.jeansebastien@gmail.com (J.-S.B.); kevim101v@gmail.com (T.-W.S.); fj888556@gmail.com (F.-C.L.); 2 Department of Materials and Textiles, Asia Eastern University of Science and Technology, New Taipei City 220303, Taiwan; fc013@mail.aeust.edu.tw; 3 Department of Materials Engineering, Ming Chi University of Technology, New Taipei City 24301, Taiwan; yyyu@mail.mcut.edu.tw

**Keywords:** supercritical carbon dioxide, dyeing, repulsion effect, end-chain length, azo dye, colorfastness

## Abstract

Supercritical carbon dioxide dyeing (SCDD) not only enables strong dyeing performance for a versatile range of polymer material but is also regarded as a green chemical media due to its low environmental impact as well as low risk of product denaturation. Over the decades, azo disperse dyes have been revealed to be efficient dyes and represent the wide majority of dyeing material. Azo dyes possess a wide variety of functional groups to optimize dye synthesis and tune the light absorption properties. Using SCDD, end-chain of different lengths, and functional group exhibiting various electronic affinity, six disperse red azo dyes were synthesized to investigate dyeing performances as woven fabric type, color strain, and color fastness after dyeing are discussed. Dye structure synthesized through a coupling reaction was confirmed by ^1^H NMR and mass spectroscopy. We found that the light absorption wavelength and absorption coefficient value variation are associated to the nature of the functional group. From the color strength values of the polyethylene terephthalate woven after dyeing, we find that the fiber host and dye dopant chemical structure greatly influence the dyeing process by providing enhanced woven, color strain, and color fastness. In comparison with commercial products, our approach not only improves the dyeing process but also guarantees a strong resistance of the dyed product against water, detergent, perspiration, abrasion, and friction.

## 1. Introduction

Conventional water dyeing leads to high intrinsic environmental problems such as waste, pollution, high energy, and water consumption. In order to overcome aforementioned drawback, carbon dioxide in supercritical conditions is facile use and exhibit high environmental protection. Supercritical carbon dioxide dyeing (SCDD) is the mainstream of dyeing due to its low environmental impact, non-toxicity, low surface viscosity, and energy, as well as low risk of product denaturation [1,2,3,4,5,6,7]. The SCDD process not only prevents polymeric swelling but also ensures effective diffusion of dye within polymer network while promoting dyestuff recovery after carbon dioxide (CO_2_) withdrawal. Through the optimization of the free energy, dissolved dye molecules form a single uniform phase with CO_2_. However, crucially, control of the temperature and pressure of the chamber to avoid low solubility and solid-phase separation between host and dye is required. As such, CO_2_ possesses a critical temperature of 31.1 °C and a critical pressure of 7.38 MPa, making it much easier to transition toward a supercritical state [1].

Recent reports focus on SCDD for synthetic and biopolymeric material such as polystyrene [8], polyester [9], cotton [4,10], polyurethane [11], wool [12], nylon [9], and polyethylene terephthalate (PET) [1,13,14]. Each polymer, as a host, possesses a different affinity with disperse dye through hydrogen bonding and hydrophobic interaction [15,16]. While polyester fiber lacks dyeing site and hydrophilic groups, its main backbone lacks extended chain side groups, leading to low dye molecule penetration kinetics [17,18]. However, PET possesses a much lower T_g_ (67–81 °C) that improves processability and wider amorphous area in comparison to other synthetic materials. Moreover, a glass transition temperature (T_g_) that is significantly lowered leads to an improved mass transfer rate within the polymeric matrix [19,20]. Conventional water dyeing process of PET fiber discharges much wastewater that is contaminated by various kinds of dispersing agents, surfactants to facilitate dye diffusion, and unused dye [21]. Therefore, using hydrophobic supercritical CO_2_ with a lower viscosity than water, fiber molecular chain free volume increases, facilitating the penetration and diffusion of disperse dyes without using auxiliary agents [22].

Disperse dyes composed of nonionic monoazo functional group and anthraquinone subunit as chromophore are widely regarded as excellent dyeing agents and are commonly synthesized from a diazonium salt and coupling intermediate [23]. Significantly, numerous azo disperse dye possesses an hydrazyl (–N=N–) group that enable to absorb light via attraction of electron resulting in strong color deepening making it suitable for dyeing applications [23,24]. Azo dispersive dyes properties are also known to be influenced by the coupling species and diazonium salt intermediate end-chain chemical group such as aromatics, aliphatics, or azo species (chromophore species). Each exhibiting unique electron withdrawing and donating strength significantly influences the dye synthesis, leading to improved color fastness [23,25]. Zhang et al. synthesized novel azo dye with aromatic hydroxyl group through a simple coupling chemical route. They show that aromatic hydroxyl group endows polyester fabric with high color fastness [26]. Hu et al. improved dyes fastness via the use of tethered azo diester and diurethane disperse dye [27]. They demonstrated that in comparison to their control product, newly synthesized dyes exhibited significantly higher molar extinction coefficient. High molar extinction coefficient is correlated to a stronger dye uptake in polymeric material as well as enhanced electron cloud pulling toward the dye chromophore group. Asiagwu et al. noticed that strong electron withdrawing hydrazyl group at the ortho position could dramatically enhance light absorption coefficient [28]. The influence of the end-group grafted onto the azo dye is expected to impact the light absorption performance, the interaction between the fiber, and the disperse dyes [29].

Herein, we synthesized various dyes series, including X019 (3-(*N*,*N*-diacetoxyethyl)amino-4-methoxyacetaniline) and X160 (3-(*N*,*N*-diethyl)aminopropionaniline). Dyes exhibit the same core chemical structure with various functional groups, such as methoxy, methyl, and nitrogen dioxide. We tune the position of the electron withdrawing and donating group and study their impact onto the chemical synthesis yield and color performances. Dye structure is determined by nuclear magnetic resonance (NMR) and mass spectroscopy (MS). The difference in light density is determined by the amount of light absorption, tuned by the chemical functional group incorporated into the azo dye host. To this end, we further fixed a methoxy group in meta-position as a strong electron withdrawing group to enhance electron pulling from the chromophore active group in synergistic effect with a methyl group at the ortho-position. To strengthen the comparison between both dye series possessing various chemical function, a dye series with a methoxy group at the ortho position of the coupling component was synthesized to evaluate the electron withdrawing effect. We have chosen PET fiber mat as the dye host due to its facile dispersive dye adsorption capabilities, excellent mechanical properties, and SCDD compatibility. Significantly, lower dyeing temperature enables the reduction of energy consumption. Considering that dyes fastness properties are crucial for dyes [30,31,32], color depth dyeing is evaluated by color strength (*K*/*S*) values based on Kubelka–Munk equation to determine the dyeing efficiency. Next, we have determined the color fastness properties for a wide range of material fabric. Consequently, dyed materials are of certain classes that match and surpass widely commercialized dye products.

## 2. Experimental Process

### 2.1. Materials

Diazo components and precursor: Aniline, 5-Methoxy-2-methyl-4-nitroaniline, 4-Nitroaniline and 2-Methoxy-4-nitroaniline were purchased from Sigma-Aldrich (St. Louis, MO, USA). All products were used as received.

Coupling component: 3-amino-4-methoxyacetanilide, 3-(*N*,*N*-diethyl)aminopropionanilide and 3-(*N*,*N*-diacetoxyethyl)amino-4-methoxyacetanilide were purchased from Sigma-Aldrich.

Other products: Sodium nitrite, sulfamic acid, sodium carbonate, isopropanol, sodium acetate, hydrochloric Acid (37%), cellulose acetate, ethanol, acetone, ethyl acetate, toluene, and n-hexane were purchased from Taiwan Green Version Technology Ltd. (New Taipei City, Taiwan) and used as received. Sodium hydroxide was purchased from Sigma-Aldrich and used as received. Thin-layer chromatographic analysis film were purchased from Macherey-Nagel Polygram^®^ SIC G/UV 254 (Düren, Germany). Carbon dioxide was purchased from Banqiao Gas Co., Ltd. (New Taipei City, Taiwan). 100% polyester fabric (knitted Polyester 75D/72F) and 100% cotton plain weave were provided by Far Eastern New Century (Taipei, Taiwan). SDC ISO standard DW multi-fiber attached cloth, white cotton cloth for AATCC friction test, ISO standard WOB lotion, and corrosion-resistant stainless-steel beads were provided by Gao Yi Enterprise Co., Ltd. (Kaohsiung City, Taiwan).

### 2.2. Preparation of Dye

#### 2.2.1. Diazonium Solution Preparation

A specified quantity of aniline-based diazo precursor and 10mL of ultrapure water are poured into a beaker, then 6 mL (37%) of hydrochloric acid are added under steady stirring and heating. Upon dissolution, a small amount of ice cubes is added to cool down the solution up to 0–5 °C. Then, 0.76 g (0.011 mole) of sodium nitrite aqueous solution is added under continuous stirring. Finally, 4-diethyl Aminobenzaldehyde (IP Solution) is used to check the presence of yellow color, and the reaction undergoes stirring until the disappearance of the yellow color, indicating complete diazotization reaction. Finally, potassium iodide (KI) test paper is used to check the presence of nitrous acid in the reaction solution. If so, traces of sulfamic acid are eliminated until the potassium iodide test paper does not change color and the solution becomes light yellow and clear, indicating a successful diazonium solution. Others diazonium solutions are prepared by replacing 4-methyl aniline with other primary aromatic species.

#### 2.2.2. Dye Fabrication and Purification

A specified quantity of coupling agent is added to the mixture of 7.5 mL cellulose acetate and 2.5 mL ultrapure water at 0–5 °C. Then, carefully drop diazonium solution into the coupling agent solution at 0–5 °C under continuous stirring until reaction’s completion. Further, an appropriate amount of sodium acetate is added to the solution to adjust the pH toward weak acidity. Following dye precipitation and filtration, the dye pH is adjusted to a neutral value with ultrapure water. After filtering, TLC is used to determine the presence of impurities in the dye. If so, impurities are washed away with ethanol followed by filtration. This step is repeated until disappearance of impurities. The formula of the developing agent was toluene: ethyl acetate: cellulose acetate = 8:2:1. For each dye, the process follows the same principle at the exception of the diazo components and coupling reagents that are selected to match the targeted dye, as shown in Table 1. Dye synthesis parameters are shown in Table S1 (see Supplementary Materials).

### 2.3. Characterization

The purified dyes were identified by FTIR, MS, and ^1^H NMR. Tetramethylsilane (TMS) was used as a reference of ^1^H NMR measurement (Bruker Advance III HD-600 MHz liquid, Bruker Daltonik GmbH, Bremen, Germany). 10mg of the dye sample was added into a flask with 1mL of CDCl_3_ to dissolve it, followed by injection into ^1^H NMR tube. Dye 019-A ^1^H NMR is given as follows, with δ (ppm): 2.3023 (9H,s,H-a.,H-F,H-i); 2.712 (3H,s,H-l.); 3.9568 (6H,s,H-n.,H-j.); 3.9659 (6H,s,H-e.,H-h.); 4.0027 (6H,t,H-d.,H-g.); 7.2013 (2H,s,H-c,H-k); 7.23 0(1H,s,H-b); 7.7568 (1H,s,H-o); 8.0029 (1H,s,H-m). Dye 019-B ^1^H NMR is given as follows, with δ (ppm): 1.9821 (3H,s,H-a); 2.1144 (3H,s,H-f,H-i); 3.9215 (3H,s,H-j); 4.1154 (6H,t,H-d,H-g); 4.4439 (2H,t,H-e,H-h); 7.2935 (1H,s,H-b,H-c,H-k); 7.9436 (1H,d,H-o,H-l); 8.3776 (1H,d,H-n,H-m). Dye 019-C ^1^H NMR is given as follows, with δ (ppm): δ2.0108 (3H,s,H-a); 2.1145 (3H,s,H-e,H-i); 3.8017 (3H,s,H-j,H-l); 4.2357 (2H,t,H-d,H-g); 4.4836 (2H,t,H-f,H-h); 7.2813 (1H,s,H-b,H-c,H-k); 7.8645 (1H,d,H-n); 8.0046 (1H,d,H-m); 8.0809 (1H,d,H-o). Dye 160-A ^1^H NMR is given as follows, with δ (ppm): 1.3018 (9H,t,H-a,H-g,H-h); 2.1082 (2H,m,H-b); 2.7164 (3H,s,H-k); 3.787 (4H,m,H-e,H-f); 3.9307 (3H,s,H-n); 6.7570 (1H,d,H-i); 7.2144 (1H,s,H-c); 7.2343 (1H,s,H-d); 7.7559 (1H,d,H-j); 7.7707 (1H,s,H-l); 8.0078 (1H,s,H-m). Dye 160-B ^1^H NMR is given as follows, with δ (ppm): 1.2544 (3H,t,H-a); 1.5760 (6H,t,H-g,H-h); 2.3177 (2H,t,H-b); 3.6571 (4H,s,H-e,H-f); 6.6976 (1H,d,H-i); 7.9959 (1H,s,H-c); 8.0911 (1H,s,H-d); 8.1696 (1H,d,H-j,H-i); 8.2092 (1H,d,H-k,H-l); 8.4009 (1H,d,H-n,H-m). Dye 160-C ^1^H NMR is given as follows, with δ (ppm): 1.2952 (3H,t,H-a); 1.3684 (6H,t,H-g,H-h); 2.5584 (2H,m,H-b); 3.9996 (4H,m,H-e,H-f); 4.0505 (5H,s,H-k); 7.1169 (1H,d,H-i); 7.660 0(2H,d,H-d); 7.6746 (2H,s,H-c); 7.7656 (1H,d,H-j); 7.8489 (2H,s,H-m); 7.8923 (3H,d,H-n); 7.9787 (1H,d,H-l). For mass spectrometer analysis of dye chemical structure, samples are first ionized through various methods carried out according to their properties. Then, 3mg to 5mg dye sample was placed in the chamber under a high magnetic field to generate a rotary motion, followed by molecular weight structure determination. The MS of dye 019-A of molecular structure C_25_H_31_N_5_O_9_: HRMS, *m*/*z*: calculated for [M^+^], 545.2 (50%) is the reference; *m*/*z*: calculated for [M–CH_3_], 515.3 (13%) display an ionic peak, possibly caused by –CH_3_ groups 2; *m*/*z*: calculated for [M–COCH_3_], 472.2 (100%) has an ionic peak similar to the reference peak, which may be caused by the –COCH_3_ group; *m*/*z*: calculated for [M–NHCOCH_3_], 194.1 (6%) has an ion peak similar to the reference peak, which may be attributed to –C_8_H_8_N_3_O_3_ Group. The ion peak of dye 019-B of molecular structure C_23_H_27_N_5_O_8_: HRMS, *m*/*z*: calculated for [M^+^], 501.2 (80%), whereas *m*/*z*: calculated for [M–2CH_3_], 471.3 (16%) has an ion peak which may originate from disruption caused by two –CH_3_ groups. Besides, *m*/*z*: calculated for [M—COCH_3_], 428.2 (100%) reference peak is caused by the –COCH_3_ group, whereas the ion peak at *m*/*z*: calculated for [M—C_6_H_4_N_3_O_2_], 150.1 (8%) is just the reference peak attributed to –C_6_H_4_N_3_O_2_ group. The ion peak of dye 019-C of molecular structure C_24_H_29_N_5_O_9_: HRMS is the reference peak at *m*/*z*: calculated for [M^+^], 531.2 (100%). The ion peak at *m*/*z*: calculated for [M–2CH_3_], 515.3 (13%) may be disrupted by two –CH_3_ groups, whereas the *m*/*z*: calculated for [M–2CH_3_–COCH_3_], 458.2 (100%) the reference peak may be caused by the interruption of the –COCH_3_ group. Finally, the *m*/*z*: calculated for [M–C_7_H_6_N_3_O_3_], 194.1 (6%) ion peak as the reference peak, which may be the –C_7_H_6_N_3_O_3_ group. The dye 160-A of molecular structure C_21_H_27_N_5_O_4_: HRMS, *m*/*z*: calculated for [M^+^], 413.2 (100%) is the reference peak; *m*/*z*: calculated for [M–CH_3_], 398.2 (70%) display an ionic peak, possibly caused by –CH_3_; *m*/*z*: calculated for [M–C_13_H_17_N_2_O], 219.1 (16%) is an ionic peak similar to the reference peak. The ion peak of dye 160-B of molecular structure C_19_H_23_N_5_O_3_: HRMS, *m*/*z*: calculated for [M^+^], 369.2 (80%), whereas *m*/*z*: calculated for [M–CH_3_], 354.2 (55%) has an ion peak which may originate from disruption caused by –CH_3_ groups. Besides, *m*/*z*: calculated for [M–C_7_H_6_N_3_O_3_], 150 (15%) is an ion peak. The dye 160-C of molecular structure C_20_H_25_N_5_O_4_: HRMS, *m*/*z*: calculated for [M^+^], 399.2 (100%) is the reference peak. *m*/*z*: calculated for [M–CH_3_], 384.2 (62%), may be caused by the disruption of one –CH_3_ group. *m*/*z*: calculated for [M–C_7_H_6_N_3_O_3_], 180.0 (13%) is an ion peak. FTIR measurements were conducted by a Fourier transform infrared spectrometers (NICOLET Is5, Thermo Fisher Scientific, Waltham, MA, USA).

### 2.4. Dyeing

The Figure S1 (see Supplementary Materials) shows the supercritical carbon experimental apparatus was used in this study. The polyester fabric of 75 D/72 F dimension was weighted at 20 g and mixed with a dye solution of 1% concentration and placed into the machine chamber. The dyeing pressure and temperature are set as follows: 3625 psi and 120 °C, respectively, for a dyeing time of 60 min.

### 2.5. Coloring Performance Characterization

The *K*/*S* colorfastness is calculated from Kubelka and Munk formula [33] and given as follows in Equation (1):

(1) $$\frac{K}{S}=\frac{{\left(1-R_{\infty }\;\right)}^{2}}{2R_{\infty }}$$

with *K* being the absorption and *S* the back-scattering coefficient, *R*_∞_ being the incident light infinite thick layer re-emission fraction. Relative coloring rate is determined as the dye cannot be exhausted during supercritical dyeing. By replacing the dyed material (continued dyeing), the dye can be exhausted. The previously obtained *K*/*S* value is divided by the first dyeing each time and accumulation of *K*/*S* value is calculated for continued dyeing. The term ${\left(\frac{K}{S}\right)}^{St}$ is the first *K*/*S* value after dyeing and $\sum_{n=1}^{\infty }{\left(\frac{K}{S}\right)}_{n}$ is the *K*/*S* value accumulation after each successive dyeing, with s and t being, respectively and given by Equation (2):

(2) $$Relative\;coloring\;rate=\frac{{\left(\frac{K}{S}\right)}^{St}}{\sum_{n=1}^{\infty }{\left(\frac{K}{S}\right)}_{n}}$$

### 2.6. Dyeing Fastness

In accordance with the: ISO 105 C06: 2010 procedure, six kinds of fibers fabric, including cellulose acetate, cotton, nylon, polyester, acrylic, and wool, were sewn to a 10 cm × 4 cm dyed cloths fabric and for all fastness tests as described below. Products were lauded into a stainless-steel bottle (volume: 550 mL, height: 125 mm, diameter: 75 mm) and placed into a washing test machine with ultrapure water, AATCC 1993 Standard Detergent (WOB), as well as 50 steel balls with a constant rotating speed of 40 ± 2 rpm. Following the washing, the sample was washed for 1 min at 40 °C with 100 mL of ultrapure water. Then, fabrics were dissembled and dried at a temperature not exceeding 60 °C.

### 2.7. Water Fastness

In accordance with the ISO 105-E01 procedure, an attached cloth of 10 cm × 4 cm was sewn with a sample cloth and dipped in ultra-pure water at room temperature for 30 min while guaranteeing uniform immersion. Then, the sample was pressed in-between two glasses to remove excess water. The sample was placed between an acrylic resin plate into a preheating test device with a specified load of 5 kg applied onto the test device. The test was conducted at 37 °C for 4 h. Following the end of the test, the load was removed, and the composite sample was extracted and dried at 60 °C.

### 2.8. Perspiration Fastness

All tests were conducted following the procedure explained above. The water was replaced with alkali or acid to mimic sweat, following the ISO 105-E04 specifications.

### 2.9. Color Fastness Abrasion and Rubbing

For dry friction, an AATCC standard white cotton cloth was clamped onto the cylindrical friction head. Then, the sample cloth was tested on the sandpaper of the machine. The downward pressure of the friction head is 9N, and it scrubbed back and forth 10 times at a speed of 1 time/second. For wet friction, a AATCC standard white cotton cloth was soaked in water at room temperature. Then, absorbent paper was used to stabilize the moisture content of the standard white cotton cloth at 65% (±5%). We clipped the standard white cotton cloth to the cylindrical friction head and placed the sample cloth to be tested on the sandpaper of the machine. The downward pressure of the friction head is 9N. During the test, it rubs back and forth 10 times at a speed of 1 time/sec. Finally, the cotton cloths of the two test methods were dried in air at a temperature not exceeding 60 °C. For all fastness tests, the result is recorded after judging the cotton cloth with gray scale or color difference.

## 3. Results and Discussion

### 3.1. Dyes Synthesis and Characterization

**Figure 1:**
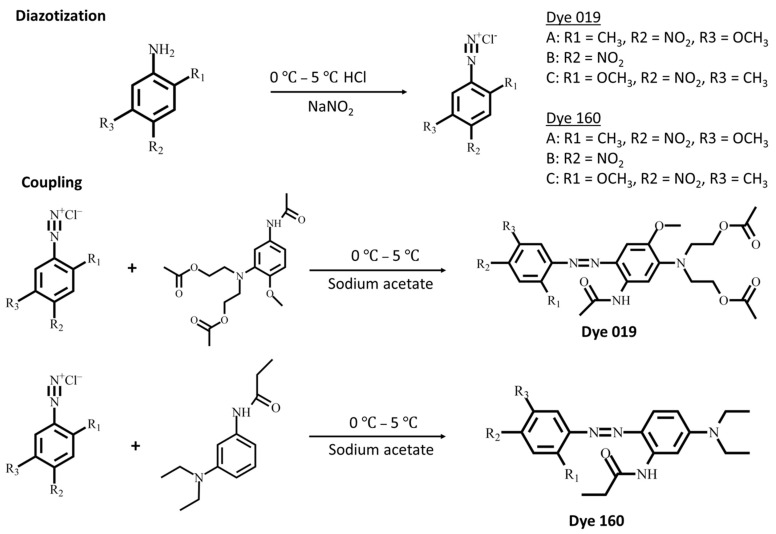
Chemical route of disperse azo dye coupling reaction and dyes formation.

In short, disperse azo dyes were obtained from a coupling process with a diazonium salt and coupling component, as shown in Figure 1 and Table 1. The diazonium salt prepared from 5-Methoxy-2-methyl-4-nitroaniline, 4-Nitroaniline, and 2-Methoxy-4-nitroaniline were synthesized through a diazotization reaction. Briefly, aromatic primary amines were used in the production of diazonium salts. The reaction temperature of diazotization was carried out at 0–5 °C to increase diazonium salt stability. At low pH, the sodium nitrite solution is more likely to dehydrate to form nitroso cations, while primary aromatic amines containing hydroxyl or sulfonic acid groups assist the diazotization reaction. The choice of the acid is also primordial to avoid formation of amino cations (NH_3_^+^), which reduces the electron density and makes it difficult to carry out nucleophilic substitution reactions. A proper amount of sodium nitrite is required to avoid formation of triazene with primary aromatic amines or quick reaction with the coupling agent producing by-products, thus reducing the diazonium salt purity.

Following the synthesis of diazonium salt precursor, six disperse azo dyes were synthesized using a coupling reaction chemical route. The dye 019 series synthesized from 3-(*N*,*N*-diacetoxyethyl)amino-4-methoxyacetaniline and dye 160 series synthesized from 3-(*N*,*N*-diethyl)aminopropionaniline coupling component with previously synthesized diazonium component. Traditionally, weakly basic amines are diazotized in acidic media, and the coupling process is required to be conducted in acidic environment for dye stability [23,26]. The coupling reaction was conducted at a temperature maintained at 0 to 5 °C to prevent instability of the diazonium salt. Under acidic conditions, –NH_2_ exhibits substantial electron-donating property, favorable to the coupling reaction. Besides working at low temperature and acidic media, diazonium salt stability is also influenced by the strength of the salt substituent electron-withdrawing group, where a stronger one will accelerate the coupling rate at the expense of the salt stability. Our dyes are composed of azo groups as the primary chromophore, combined with multiple auxochromic groups to endow the dye with a particular color and dyeing capability. Interestingly, the presence of the methoxy group at the meta-position originates from the nature of coupling agent substituents and pH of the media. Electron withdrawing groups weaken the electron density, whereas electron repulsive groups enhance the electron density toward the coupling agent and favor reactions at the meta-position of the substituent. More details are given about the synthesis principle and objectives are given in the Supplementary Materials. As shown in Figure 1, three sub-series of dye 019 and 160 series were synthesized by tuning the position of the electron withdrawing and repulsive group to endow disperse dye with specific properties, including high light absorption and strong color fastness.

The structural analysis of dye 019 and dye 160 are given by the ^1^H NMR spectroscopy illustrated in Figures S2–S8 (see Supplementary Materials). First, the structure of dye 019-A from ^1^H NMR spectroscopy data shows that there are nine double peaks of hydrogen at the position δ2.3023 attributed to the hydrogen of acetyl group a, f, and I of the dye. Furthermore, three single peaks of hydrogen with a chemical shift of δ2.712 are attributed to the hydrogen of the methyl l of the dye. Six single peaks on δ3.9568 and δ3.9659 correspond to the carbon j, e, and h, respectively. At the position of chemical shift δ4.0027, six three-wave peaks of hydrogen ascribed to the carbon number d and g of the dye. Two single peaks of hydrogen at the position of chemical shift δ7.2013 correspond to the carbon number c and k of the dye. Single hydrogen peak at δ7.230 is associated to the nitrogen b, there is a single peak of hydrogen at the position of chemical shift δ7.7568, which is judged to be the hydrogen on the carbon of dye o at the position of chemical shift δ8.0029. Finally, there is a single peak of hydrogen, which is judged to be the hydrogen on the m carbon of the dye. ^1^H NMR studies of other dyes can be found in the Table S2 (see Supplementary Materials) according to the dye lettering presented in the Figure S2 (see Supplementary Materials).

Figures S9–S14, Table S3, and Table S4 (see Supplementary Materials) show the mass spectroscopy analysis of dye 019 and 160 series. As shown in Figures S9–S11 (see Supplementary Materials), the dye 019-A of molecular structure C_25_H_31_N_5_O_9_: HRMS, *m*/*z*: calculated for [M^+^], 545.2 (50%) is the reference; *m*/*z*: calculated for [M–CH_3_], 515.3 (13%) display an ionic peak, possibly caused by –CH_3_ groups 2; *m*/*z*: calculated for [M–COCH_3_], 472.2 (100%) has an ionic peak similar to the reference peak, which may be caused by the –COCH_3_ group; *m*/*z*: calculated for [M–NHCOCH_3_], 194.1 (6%) has an ion peak similar to the reference peak, which may be attributed to –C_8_H_8_N_3_O_3_ Group. The ion peak of dye 019-B of molecular structure C_23_H_27_N_5_O_8_: HRMS, *m*/*z*: calculated for [M^+^], 501.2 (80%), whereas *m*/*z*: calculated for [M–2CH_3_], 471.3 (16%) has an ion peak which may originate from disruption caused by two –CH_3_ groups. Besides, *m*/*z*: calculated for [M–COCH_3_], 428.2 (100%) reference peak is caused by the –COCH_3_ group whereas the ion peak at *m*/*z*: calculated for [M–C_6_H_4_N_3_O_2_], 150.1 (8%) is just the reference peak attributed to –C_6_H_4_N_3_O_2_ group. The ion peak of dye 019-C of molecular structure C_24_H_29_N_5_O_9_: HRMS is the reference peak at *m*/*z*: calculated for [M^+^], 531.2(100%). The ion peak at *m*/*z*: calculated for [M–2CH_3_], 515.3 (13%) may be disrupted by two –CH_3_ groups whereas the *m*/*z*: calculated for [M–2CH_3_–COCH_3_], 458.2 (100%) the reference peak may be caused by the interruption of the –COCH_3_ group. Finally, the *m*/*z*: calculated for [M–C_7_H_6_N_3_O_3_], 194.1 (6%) ion peak as the reference peak, which may be the –C_7_H_6_N_3_O_3_ group. As shown in Figures S12–S14 (see Supplementary Materials), the dye 160-A of molecular structure C_21_H_27_N_5_O_4_: HRMS, *m*/*z*: calculated for [M^+^], 413.2 (100%) is the reference peak; *m*/*z*: calculated for [M–CH_3_], 398.2 (70%) display an ionic peak, possibly caused by –CH_3_; *m*/*z*: calculated for [M–C_13_H_17_N_2_O], 219.1 (16%) is an ionic peak similar to the reference peak. The ion peak of dye 160-B of molecular structure C_19_H_23_N_5_O_3_: HRMS, *m*/*z*: calculated for [M^+^], 369.2 (80%) whereas *m*/*z*: calculated for [M–CH_3_], 354.2 (55%) has an ion peak which may originate from disruption caused by –CH_3_ groups. Besides, *m*/*z*: calculated for [M–C_7_H_6_N_3_O_3_], 150 (15%) is an ion peak. The dye 160-C of molecular structure C_20_H_25_N_5_O_4_: HRMS, *m*/*z*: calculated for [M^+^], 399.2 (100%) is the reference peak. *m*/*z*: calculated for [M–CH_3_], 384.2 (62%), may be caused by the disruption of one-CH_3_ group. *m*/*z*: calculated for [M–C_7_H_6_N_3_O_3_], 180.0 (13%) is an ion peak.

**Figure 2:**
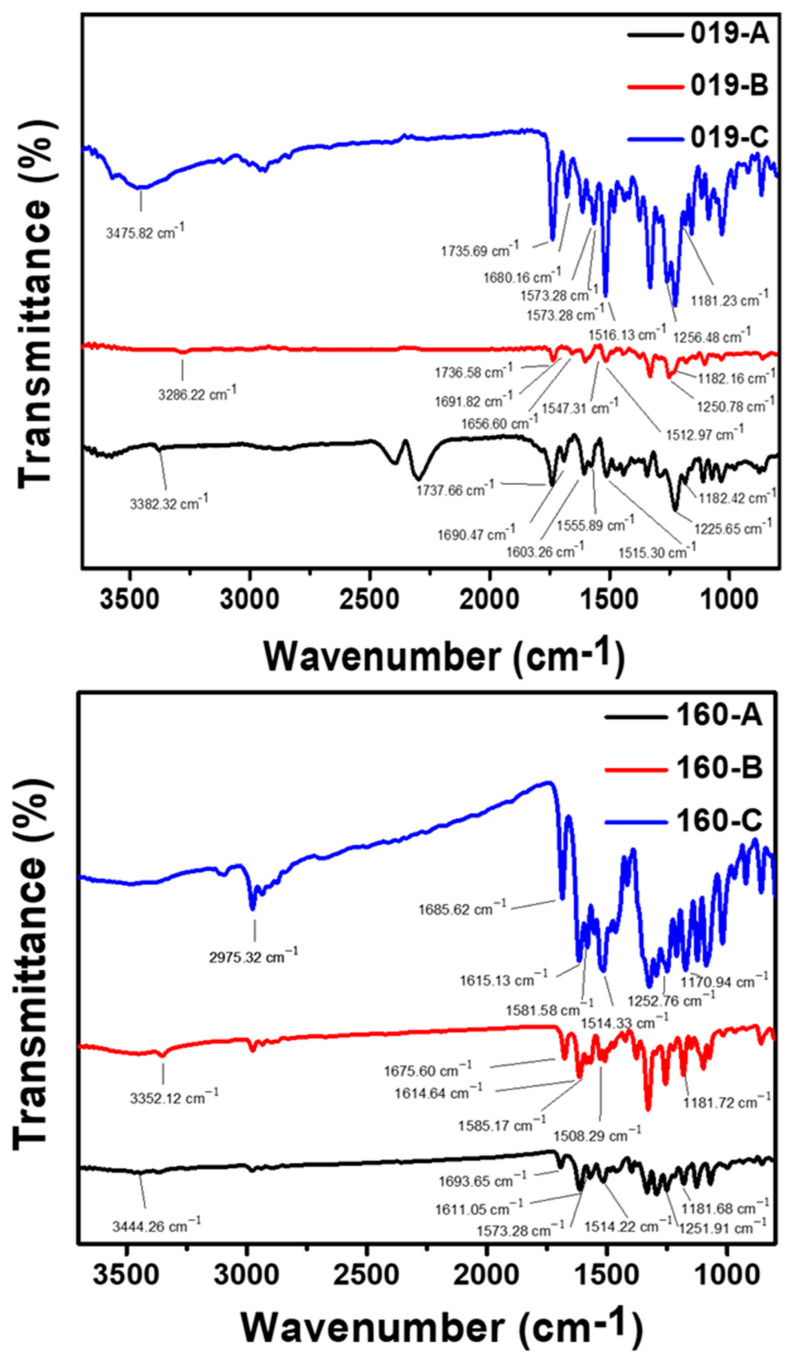
FTIR spectra of synthesized 019 and 160 series dyes.

Further, we conducted FTIR, as shown in Figure 2, for clarity, all values are given for the following dye order 019-A, 019-B, and 019-C. We find that for the dye series 019, all dyes present a signal at 3382.32, 3286.22, and 3475.82 cm^−1^, respectively, corresponding to the –N–H– stretching of the coupling component. Furthermore, the signal at 1555.89, 1547.31, and 1565.46 cm^−1^, respectively, is ascribed to the –N=N– of the chromophore. A –C–H– signal was observed at 1603.26, 1656.60, and 1573.28 cm^−1^, respectively. Simultaneously, the ester function of the long chain of the coupling component was observed with a –C=O– stretching at 1737.66, 1736.58, and 1735.69 cm^−1^, respectively. The nitro function of the diazonium salt precursor (–N–O– stretching) was observed at 1515.30, 1512.97 and 1516.13 cm^−1^, respectively. A –C–O– stretching, characteristic of methoxy group, was observed at 1225.65, 1250.78, and 1256.48 cm^−1^, respectively. The –C–N– of the coupling component was observed at 1182.42, 1182.16, and 1181.23 cm^−1^, respectively. Finally, the amide function with the –C=O– stretching signal peak was observed at 1690.47, 1691.82, and 1680.16 cm^−1^, respectively. Dye series 160 was also characterized by FTIR and all dyes present a signal at 3444.26, 3352.12, and 2975.32 cm^−1^, respectively, corresponding to the –N–H– stretching of the coupling component. Furthermore, the signal at 1611.05, 1614.64, and 1615.13 cm^−1^, respectively, is ascribed to the –N=N– of the chromophore. A –C–H– signal was observed at 1573.28, 1585.17, and 1581.58 cm^−1^, respectively. Simultaneously, the amide function of the of the coupling component was observed with a –C=O– stretching at 1693.65, 1675.60, and 1685.62 cm^−1^, respectively. The nitro function of the diazonium salt precursor (–N–O– stretching) was observed at 1514.22, 1508.29, and 1514.33 cm^−1^, respectively. A –C–O– stretching, characteristic of methoxy group, was observed at 1251.91 and 1252.76 cm^−1^, for dye 160-A and 160-C, respectively. Finally, the –C–N– of the coupling component was observed at 1181.68, 1181.72, and 1170.94 cm^−1^, respectively [34,35].

In conclusion, our dyes display highly satisfying chemical properties while having high purity and yield. Therefore, with dye structure integrity being demonstrated, the presence of functional group at the desired position is expected to significantly influence the optical properties of disperse dyes.

### 3.2. Optical Characterization of Dyes

Synthesized on a D-π-A model with electron-donating (D) or electron-accepting (A) functional groups, disperse dyes exhibit various color deepness, strength, and shades in correlation with the strength of the electron withdrawing group. The disperse dyes were dispersed with ethanol at a concentration of 10^−5^ g/mL for UV–Vis experiment with the maximum absorption wavelength (λ max) and absorption coefficient (ε) are shown in the Table 2.

**Figure 3:**
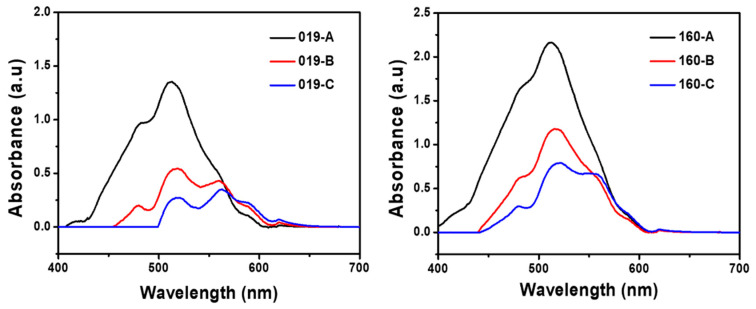
UV–Vis spectra of synthesized 019 and 160 series dyes.

As shown, in Figure 3, the maximum absorption wavelength is ranging around the same maximum region around 513–562 nm thus emitting red light, differences are observed due to the introduction of end-group grafted onto aromatics group and side-chains of different length. The maximum absorption wavelengths of dyes 019-A and 160-A are 513 nm, 512 nm, respectively, the maximum absorption wavelengths of dye 019-B and 160-B are 519 nm and 516 nm, and the maximum absorption wavelengths of dye 019-C and 160-C are 562 nm and 516 nm, respectively. The low light absorption wavelength shift means that the length of the side-chains does not significantly influence on the maximum absorption wavelength. Besides, for each dye series, ε value are over 35,000 L·mol^−1^·cm^−1^ up to 216,000 L·mol^−1^·cm^−1^. It is admitted that a higher ε value is associated with a lower number of dyes required to dye fabric as well as a much higher electron repulsion effect [27]. Zhang et al.’s approach led to an ε to be as high as 31,000 L·mol^−1^·cm^−1^. While the hydroxyl group endows dye with water compatibility, it is expected that the hydroxyl group being a weak electron withdrawing group reduces dyeing efficiency [26]. By replacing hydroxyl with stronger electron withdrawing group such as ester and urethane group, the hypsochromic shift increase, translating a stronger electron pulling effect [27]. Furthermore, significant variations of ε are observed within and between dyes series, with the dyes 160 series exhibiting the highest ε value overall. First, the methyl group at the aromatic ring para position acts as an electron, donating substituent pushing electrons toward the chromophore. Such a repulsive effect causes the bandgap to widen in energy, translating a shift to the shorter wavelength of 4–6 nm. Second, high ε is attributed to the electron withdrawing methoxy group at the meta of the aromatic ring so that the electron pulling effect from the amine group is enhanced for dyes series A.

This is confirmed, as dye 019-C and dye 160-C maximum wavelength of absorption has drastically shifted toward the red region, as they do not possess methoxy group at the meta-position of the phenyl ring nor methyl group. According to Cinar et al. [36], azo disperse dye constructed on –C–N=N–C structure with phenyl rings has been observed to have allowed or forbidden transitions due to symmetry consideration. Cinar et al. not only assigned their azo disperse transition at 490 nm to n-π* but also highlighted that the band-gap of the chromophore could be decreased with increased π-electron delocalization. However, Lee et al. highlighted that the presence of a methoxy group onto the coupling component at the meta-position induces a bathochromic and hypochromic effect [37]. Therefore, the electron pushing effect is weakened. Herein, the molecule functional group engineering results in a red-shifting of the absorbance while preserving the n-π* transition, with the n orbital corresponding to the lone pair of electrons of the N atoms. Our observation corroborates this statement as our dye series 019 possesses a methoxy group at the meta-position of the coupling component. Dye 019 series wavelength shifts much more significantly toward red region with lower ε value in comparison to the dye 160 series. This observation highlights the importance of the methoxy group at the meta-position onto the aromatic group with respect to the D-π-A dye model to significantly enhance the light absorption performances. Overall, all dyes are suitable for dyeing.

### 3.3. Dyeing Performances

#### 3.3.1. Apparent Colorfastness K/S

Conventional water polyester fiber dyeing with disperse dyes requires pre-treatment and being coated with auxiliary agents (such as methyl salicylate, benzoate, chlorobenzene, methyl naphthalene). On the contrary, SCDD with disperse dye does not require pre-treatment and can be dyed simply by drying and grinding. Disperse dyes are hardly soluble in water, and are more likely to be dispersed in supercritical CO_2_ while avoiding phase separation. Therefore, the crystallinity ratio of the PET fibers directly impacts the dye penetration efficiency into the PET fibers [38,39]. Moreover, CO_2_ does not penetrate the PET fabric due to hydrophobicity incompatibility, therefore facilitating dye diffusion within the amorphous PET part. During dyeing, the dye is first absorbed onto the fiber boundary layer during dye flowing, followed by inward diffusion within the fiber boundary layer, triggering intermolecular interaction between fiber host and dye. The diffusion rate is proportional to the temperature and dye concentration. Therefore, increasing the dyeing temperature increases the pores in the amorphous area of the polyester fiber and reduces the resistance of the dye molecules to diffuse into the fiber, thereby increasing the dye uptake rate and reducing the diffusion time. One of the main advantages of PET fibers is its low T_g_ facilitating chain motion and wider amorphous area.

The PET fabric was mixed with dye solution at 1% concentration and placed into the machine chamber, followed by a SCDD process for 60 min at 120 °C. A high-temperature process facilitates the penetration of dyes within non-crystalline area of PET fibers. To assess the dyeing performances, the dye absorption efficiency of dyed PET fabric is calculated using the *K*/*S* formula. We find that our dyes *K*/*S* apparent color density range from 14.78 (dye 019-C) to 20.02 (dye 160-A). Table 3 shows that dye 019-B has a higher apparent color concentration in the 019 series (16.376), and relative coloring rate is 89.30%, higher than other 019 series dyes. The apparent color concentration of dye 160-A is higher (20.02), and its first relative coloring rate is 88.82% better than other 160 series dyes and 019-C has the highest first relative coloring rate among all dyes. Based on the above description, 019-A, 019-B, 019-C, 160-A are more suitable for SCDD dyeing than other dyes of their corresponding series. We find that the methoxy group, as shown previously, also significantly impacts the apparent color density by improving the electron pulling effect of successfully incorporated dye into PET. Disperse azo dye with strong electron withdrawing groups provide a higher electron pulling effect, leading to improved color fastness [27]. It shows that the dye’s molecular structure directly impacts the color rendering yield following the dyeing process. Moreover, it is known that the addition of side chain of significant length such as alkyl chains to the dye can increase the apparent color density, the first relative coloring rate, and influence the colorfastness properties after dyeing [40,41]. Freeman et al. [39] demonstrated that the length of the alkyl chains attached to the phenyl ring do not exhibit a significant influence onto the polylactide fibers dyeing performances but greatly influence the dyeing fastness performances. Considering that the dye 019 series possess longer end-chain compared to the dye 160 series, the difference in color fastness originates from the excessive length of the carbon chains, reducing penetration and diffusion of dye into the amorphous area of the polyester fiber. As a result, both the apparent color density and the first relative coloring rate significantly decrease. Therefore, the dye’s solubility in supercritical CO_2_ is enhanced through the introduction of an aniline component for the synthesis of diazo-based disperse dye. However, long side chains are likely to mitigate the dye diffusion within a non-crystalline area of the fiber, as increasing the length of the long-chain group makes the dye molecules larger.

#### 3.3.2. Color Fastness Properties

Different textile fibers fabrics have repeatedly been exposed to various environment in order to evaluate the dyes resilience within fibers fabric in accordance with suitable ISO procedure (We refer to each table description for the ISO procedure name). We have used cotton, nylon, polyester, wool, acrylic, and cellulose acetate fabric sewed onto the dyed stuff. According to ISO test procedure regulation, the washing and dyeing fastness scale range from 1 to 5, with 4–5 associated with excellent wash fastness output. In brief, fabrics were sewn together before being exposed to detergent, water, alkali, and acidic environments. As shown in Table S5 (see Supplementary Materials), the color fastness to washing of dyes 160-A, 160-B, and 160-C are grade 2 when attached to the nylon cloth, followed by exposition to a standard detergent. Dyes 160-A and 160-B reach grade 2–3 when sewed with cellulose acetate mat. The washing fastness of other dyes is above grade 3–4, which meets the commercial standard. Water washing and color fastness has also been investigated, and fabric was exposed to ultrapure water before being dried following ISO 105 E01:2010 specification, as shown in Table 4. The fastness is grade 3 or above for any sewed dyed fabric, which also meets the commercial standard. To go further, we have assessed the dyed fabric resistance to perspiration through exposition to acid (Table 5) and alkali (Table S6, see Supplementary Materials) environments. The observed color fastness after dyeing is all above grade 4, which is in line with commercial standards for any of the fibers and environmental exposure. Finally, dyeing fastness to abrasion and rubbing was investigated and all dyes are above 4, which meets commercial standards as shown in Table 6. We find that regardless of the chemical structure that impact the dyes uptakes and performances, our dyes are strongly impregnated into our fabrics and strongly protected against external degradation.

## 4. Conclusions

In this study, we have successfully synthesized six disperse azo dyes through a facile chemical route consisting of using a coupling component with a dye precursor. We confirmed the dyes structure by ^1^H NMR and MS. Following synthesis, dyes absorption color was confirmed to be red. Not only did the end-chains length only weakly influence the color absorption, but the ortho-position of the methoxy group onto the aromatic group strengthened the electron repulsion effect, therefore granting our dyes with high molar extinction coefficient. Further, the dyes 019-A, 019-B, 019-C, and 160-A exhibit the best PET fabric dyeing performances using supercritical CO_2_. According to the apparent color concentration of polyester fabric after dyeing, aniline increases the dyes solubility in supercritical CO_2_, while excessive end-chain length negatively impacts dye performances. Detergent, water, alkali, and acid color fastness test were performed and highlight the superior performances of our synthesized dyes. Abrasion and rubbing test similarly highlight the high diffusion of dye within PET fabric and confirm our dyes meet commercial standard.

## Figures and Tables

**Table 1:** The yield and general structure of synthesized dye series.

Dye	R_1_	R_2_	R_3_	General Structure	Mass	Molecular Formula	Yield
019-A	CH_3_	NO_2_	OCH_3_	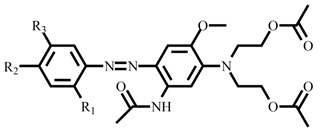	545.21	C_25_H_31_N_5_O_9_	87%
019-B	-	NO_2_	-		501.19	C_23_H_27_N_5_O_8_	84.4%
019-C	OCH_3_	NO_2_	-		531.20	C_24_H_29_N_5_O_9_	83.7%
160-A	CH_3_	NO_2_	OCH_3_	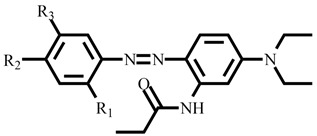	413.21	C_21_H_27_N_5_O_4_	81.5%
160-B	-	NO_2_	-		369.18	C_19_H_23_N_5_O_3_	78.7%
160-C	OCH_3_	NO_2_	-		399.19	C_20_H_25_N_5_O_4_	83.2%

**Table 2:** Optical properties of series dyes.

Dye	Maximum Absorption Wavelength (nm)	Absorbance	Absorption Coefficient (L·mol^−1^·cm^−1^)	Log(e)
019-A	513	1.353535	135354	5.131
019-B	519	0.544376	54437.6	4.736
019-C	562	0.351125	35112.5	4.545
160-A	512	2.162309	216231	5.335
160-B	516	1.178727	117873	5.071
160-C	522	0.792503	79250.3	4.899

**Table 3:** Apparent color concentration and first time dyeing relative color of series dyes following supercritical carbon disperse dye dyeing.

Dye	Apparent Color Density	Relative Color First Time
019-A	15.5170	89.49%
019-B	16.3760	89.30%
019-C	14.7890	90.15%
160-A	20.02	88.82%
160-B	18.9330	68.92%
160-C	16.7450	72.78%

**Table 4:** Dyeing fastness to water after dyeing according to the ISO 105 E01:2010 protocol.

Dye	Cellulose Acetate	Cotton	Nylon	Polyester	Acrylic	Wool
019-A	3–4	4	3–4	4–5	5	4–5
019-B	4–5	4–5	4	4–5	4–5	4–5
019-C	4–5	4–5	4	4–5	5	5
160-A	4–5	4–5	4	4–5	5	5
160-B	4–5	4	4	4	5	4–5
160-C	4–5	5	4–5	4–5	5	5

**Table 5:** Color fastness in acidic media after dyeing according to the ISO 105 E04:2008 protocol.

Dye	Cellulose Acetate	Cotton	Nylon	Polyester	Acrylic	Wool
019-A	4–5	4–5	4	4–5	4–5	4–5
019-B	4	4–5	4	4–5	5	4–5
019-C	4–5	4–5	4	4–5	5	4–5
160-A	4–5	4–5	4	4–5	5	5
160-B	4–5	4–5	4–5	4–5	5	5
160-C	4–5	4–5	4–5	4–5	5	5

**Table 6:** Color fastness to abrasion and rubbing after dyeing according to the AATCC 8 protocol.

Dye	Dry Friction	Wet Friction
019-A	4	4–5
019-B	4	4–5
019-C	4	5
160-A	4	4–5
160-B	4	4–5
160-C	4	4–5

## Data Availability

The data presented are contained within the article.

## Supplementary Materials

The following supporting information can be downloaded at: https://www.mdpi.com/article/10.3390/polym14153020/s1, Figure S1: Supercritical carbon installation; Figure S2: Dye lettering for NMR characterization; Figure S3: NMR analysis of dye 019-A in CDCl_3_; Figure S4: NMR analysis of dye 019-B in CDCl_3_; Figure S5: NMR analysis of dye 019-C in CDCl_3_; Figure S6: NMR analysis of dye 160-A in CDCl_3_; Figure S7: NMR analysis of dye 160-B in CDCl_3_; Figure S8: NMR analysis of dye 160-C in CDCl_3_; Figure S9: Mass spectroscopy analysis of dye 019-A; Figure S10: Mass spectroscopy analysis of dye 019-B; Figure S11: Mass spectroscopy analysis of dye 019-C; Figure S12: Mass spectroscopy analysis of dye 160-A; Figure S13: Mass spectroscopy analysis of dye 160-B; Figure S14: Mass spectroscopy analysis of dye 160-C; Table S1: Experimental parameters of disperse dye synthesis; Table S2: Disperse dye series NMR analysis; Table S3: Mass spectrum analysis of dye 019 series; Table S4: Mass spectrum analysis of dye 160 series; Table S5: Washing fastness to detergent after dyeing according to the ISO 105 C06:2010 protocol; Table S6: Color fastness in alkali media after dyeing according to the ISO 105 E04:2008 protocol.
