# Supplementary material for: Synthesis of Azo Disperse Dyes with High Absorption for Efficient Polyethylene Terephthalate Dyeing Performances in Supercritical Carbon Dioxide
Source: Polymers (Basel). 2022 Jul 26;14(15):3020. doi: 10.3390/polym14153020 (PMC9331285; doi:10.3390/polym14153020)
Supplement: Supplementary file 1 [file polymers-14-03020-s001.zip › polymers-1795551-supplementary.pdf]

polymers MDPI

# Supplementary Materials: Synthesis of Azo Disperse Dyes with High Absorption for Efficient Polyethylene Terephthalate Dyeing Performance in Supercritical Carbon Dioxide

**Yu-Wen Cheng [1,†], Jean-Sebastien Benas [1,†], Fang-Cheng Liang [1,*], Shang-Ming Lin [2], Ting-Wang Sun [1], Fu-Chieh Liu [1], Yang-Yen Yu [3] and Chi-Ching Kuo [1,*]**

1 Institute of Organic and Polymeric Materials, Research and Development Center of Smart Textile Technology, National Taipei University of Technology, No. 1, Sec. 3, Chung-Hsiao East Road, Taipei 10608, Taiwan; weven007@yahoo.com.tw (Y.-W.C.); benas.jeansebastien@gmail.com (J.-S.B.); kevim101v@gmail.com (T.-W.S.); fj888556@gmail.com (Fu-Chieh Liu)
2 Department of Materials and Textiles, Asia Eastern University of Science and Technology, New Taipei City 220303, Taiwan; fc013@mail.aeust.edu.tw
3 Department of Materials Engineering, Ming Chi University of Technology, New Taipei City 24301, Taiwan; yyyu@mail.mcut.edu.tw
* Correspondence: frank62112003@mail.ntut.edu.tw (Fang-Cheng Liang); kuocc@mail.ntut.edu.tw (C.-C.K.); Tel.: +886-2-27712171 (ext. 2407); Fax: +886-2-27317174
† Y.-W. Cheng and J.-S. Benas contributed equally to this work.

## 1.Principle and Details of Disperse Azo Dyes Synthesis

### *1.1. Dye Color Theory*

Dyes absorb part of the visible light to re-emit complementary colors. For dyes of the same chemical structure, the light density gradient is determined by the amount of light absorption that induces a darker color with higher amounts of absorbed light. Unsaturated groups, which are also called chromophores, are at the origin of a dye's light sensitivity, and molecules containing chromophores must contain specific group such as nitro, azo, carbonyl, nitroso, vinyl, thiocarbonyl, and methine groups. The color can be darkened by increasing the number of conjugated double bonds. However, not all groups are chromophores, such as conjugated carbon chains and aromatic groups. The introduction of other substituent groups may influence a dye's behavior, as it can donate or withdraw electrons from the chromophore center. For instance, amine groups, called autochromes, can deepen the color and improve dyeing performance.

### *1.2. Principles of Azo Dye Synthesis [1–7]*

Azo dye molecules contain one or more azo groups (hydrazyl) (–N=N–). This type of dye uses azo groups as the primary chromophore combined with multiple auxochromic groups (hydroxyl, amino, halogen, sulfonic acid group, etc.) or other chromophores to endow the dye with a particular color and dyeability.

#### 1.2.1. Diazotization Reaction

First, the primary aromatic amine reacts with nitrous acid to form a diazonium salt followed by a coupling reaction with the coupling agent to produce azo dyes. Since nitrous acid is very easy to decompose, sodium nitrite and mineral acids (hydrochloric acid, sulfuric acid) are usually used in the reaction. Nitrous acid decomposes into $NO^+$ or $N_2O_3$ under acidic conditions to further carry out nucleophilic substitution reactions with compounds with a high electron density, such as primary aromatic amines. The diazotization reaction is as follows:

Protonation of nitrous acid:

$$HO-N=O + H^{+} \longrightarrow H_2\overset{+}{O}-N=O$$

Water removal to produce electrophilic nitroso ions:

$$H_2\overset{+}{O}—N{=}O \longrightarrow [N{=}O]^+ + H_2O$$

Aromatic primary amine nucleophilic attacks toward the nitroso cation:

$$[N{=}O]^+ + Ar—NH_2 \longrightarrow Ar—\overset{+}{NH_2}—NO$$

Deprotonation and rearrangement to obtain diazonium hydroxide:

$$Ar—\overset{+}{NH_2}—NO \longrightarrow Ar—N{=}N—OH + H^+$$

The oxygen atom is protonated, leading to the formation of a diazonium cation:

$$Ar—\overset{+}{NH_2}—NO \longrightarrow Ar—N{=}N—OH + H^+$$

Generally, aromatic primary amines are used in the production of diazonium salts. Upon the reaction of a secondary aromatic amine, only nitrosamine can be formed. In the case of using an aliphatic primary amine as a reactant, the formation of a diazonium salt after reacting with nitrous acid is possible; however, no resonance phenomenon is observed, making the salt unstable. Once formed, it is hydrolyzed immediately and cannot be coupled with the coupling agent. Generally, the factors affecting diazotization include the following factors:

Reaction temperature: As the diazotization reaction is an exothermic reaction, the reaction temperature of diazotization should be carried out at 0–5 °C to increase diazonium salt stability and to prevent nitrous acid gas from overflowing.

pH: At high pH levels, primary aromatic amines containing hydroxyl or sulfonic acid groups are insoluble in water and suppress the diazotization reaction. At low pH levels, the sodium nitrite solution is more likely to dehydrate to form nitroso cations. The choice of the acid is also primordial to avoid the formation of amino cations ($NH_3^+$) on the relatively aromatic primary amines. This type of group reduces the electron density and makes it difficult to carry out nucleophilic substitution reactions, which reduces the rate of diazotization reactions.

Dosage of sodium nitrite: Sodium nitrite forms nitroso compounds in an acidic solution and then undergoes a diazotization reaction with primary aromatic amines. A proper amount of sodium nitrite is required to avoid the formation of triazene with primary aromatic amines or to avoid it from quickly reacting with the coupling agent to produce by-products, thus reducing the purity of the diazonium salt. Therefore, during the diazotization reaction, adding sulfamic acid or urea in the appropriate amounts can remove residual nitrous acid. The reaction method is as follows:

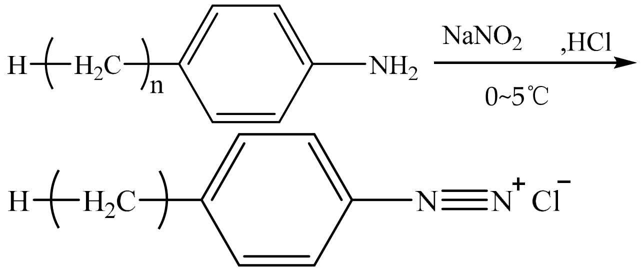


1.2.2. Coupling reaction

The diazonium salt obtained after the diazotization reaction can undergo an electrophilic substitution reaction with coupling components such as phenols, amines, or their derivatives to form an azo compound.

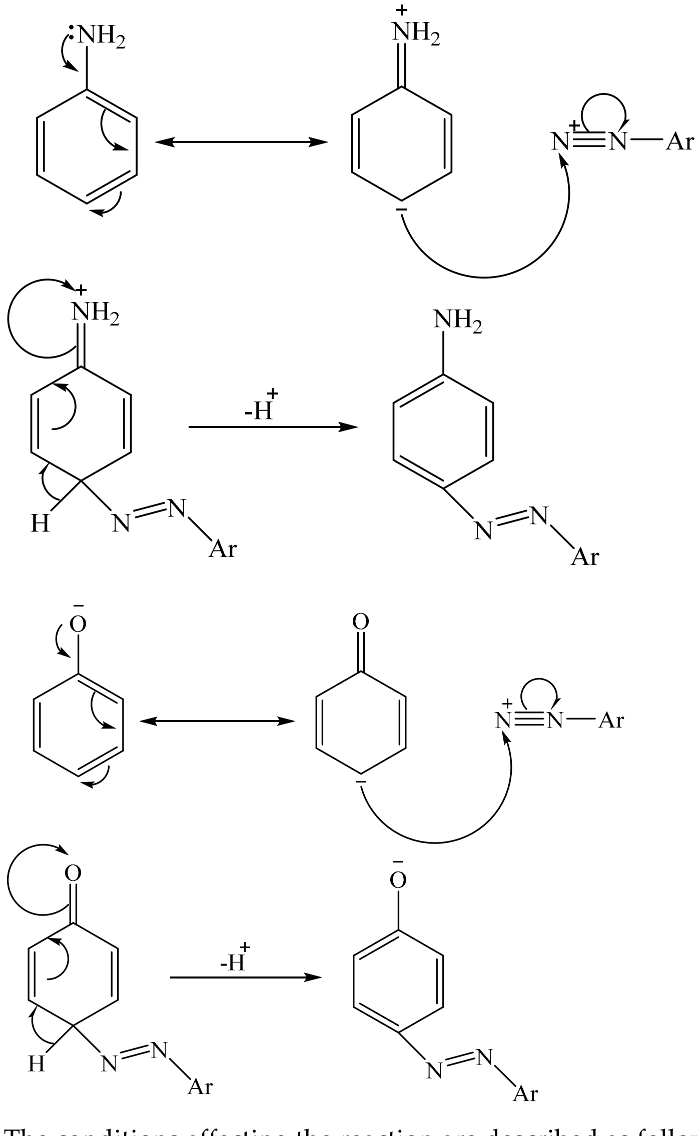

The conditions affecting the reaction are described as follows:

Reaction temperature: Most diazonium salts are unstable, leading to decomposition during the coupling reaction. Upon the reaction temperature increasing by 10 °C, the coupling reaction rate will increase by 2–2.4-fold, but the decomposition rate of the diazonium salt will increase 3.1–5.3-fold. Therefore, it is not suitable to raise the temperature to increase the coupling reaction rate. The general reaction temperature is conducted from 0 °C to 5 °C to avoid increasing the instability of the diazonium salt and its decomposition rate, neither of which are favorable to the coupling overall.

Substituents of the diazonium salt: The electron-withdrawing group in the diazonium salt simultaneously increases the strength of the positive charge on the diazonium salt and accelerates the coupling rate. Increased diazonium salt decomposition kinetics are also observed. On the contrary, the electron-donating substituent will reduce the reactivity, but the diazonium salt is more stable.

Substituents of the coupling agent: The coupling reaction is an electrophilic substitution reaction, so the coupling agent will affect the speed and position of the substituent. For instance, an electron-withdrawing group substituent weakens the electron cloud density on the coupling agent, reducing the electrophilic substitution reaction rate. On the

contrary, an electron-donating substituent increases the electron cloud density toward the coupling agent, and the coupling reaction is more likely to occur in the para or ortho position of the substituent.

pH: The coupling reaction is affected by the pH of the media. For instance, using aromatic alcohols (such as phenols, etc.) as a coupling compound requires that the media set under alkaline conditions due to –OH forming –O– under alkaline conditions. The coupling reaction is facilitated as the electron-donating effect is enhanced. On the contrary, an aromatic amine (such as aniline, etc.) requires acidic conditions. In acidic media, $–NH_2$ has substantial electron-donating properties that are favorable to the coupling reaction. Since $–SO_3H$ produces a steric effect, the reaction at the para position of the substituent is suppressed, forcing the reaction to occur at the ortho position of the substituent.

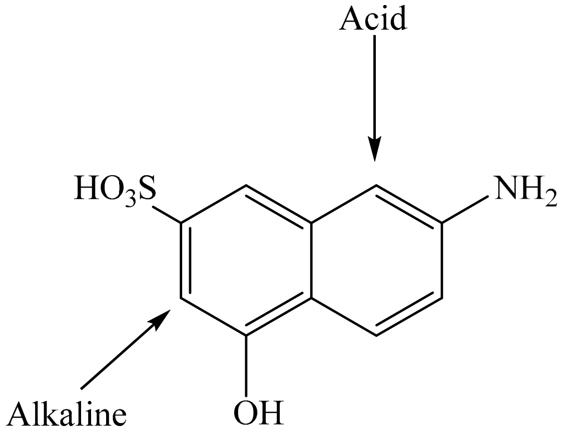

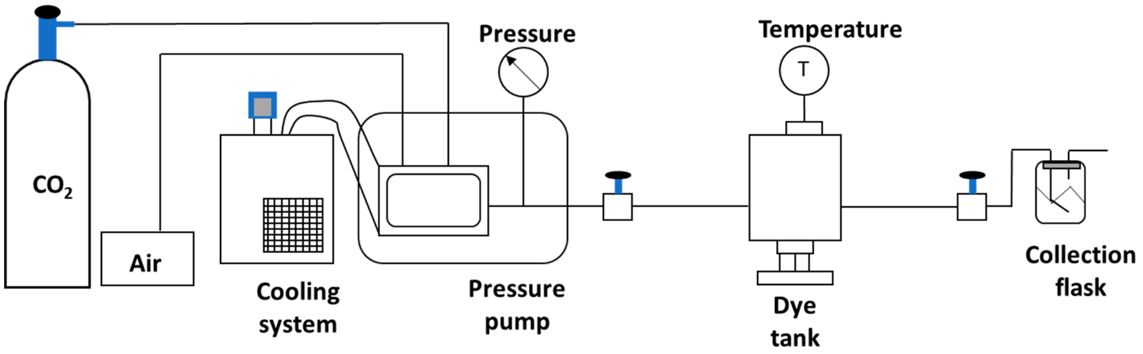


**Figure S1.** Supercritical carbon installation.

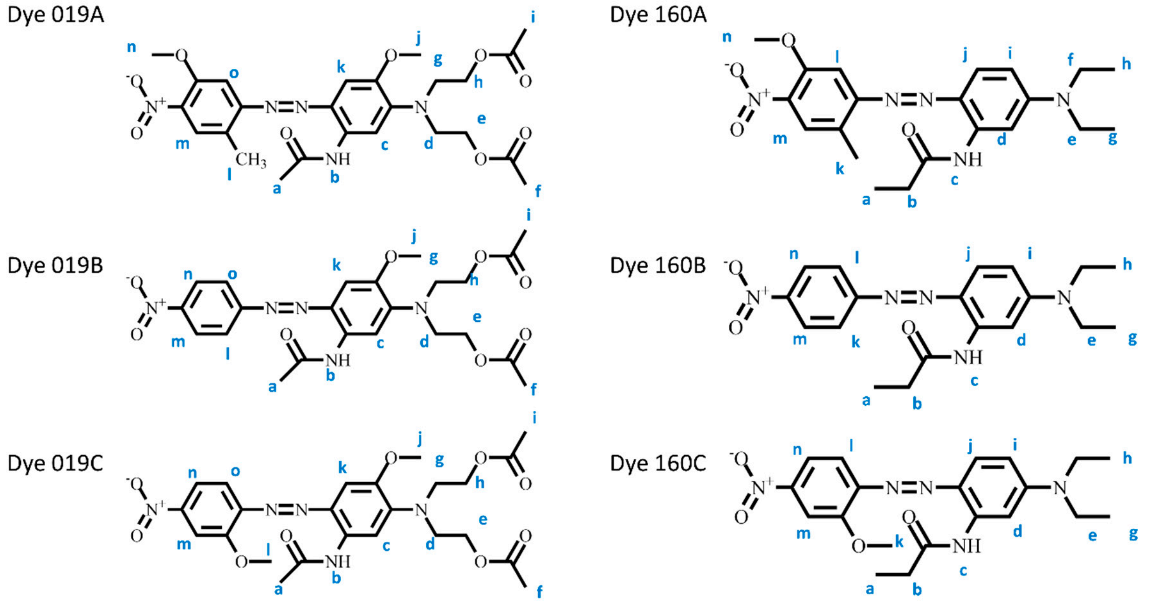


**Figure S2.** Dye lettering for NMR characterization.

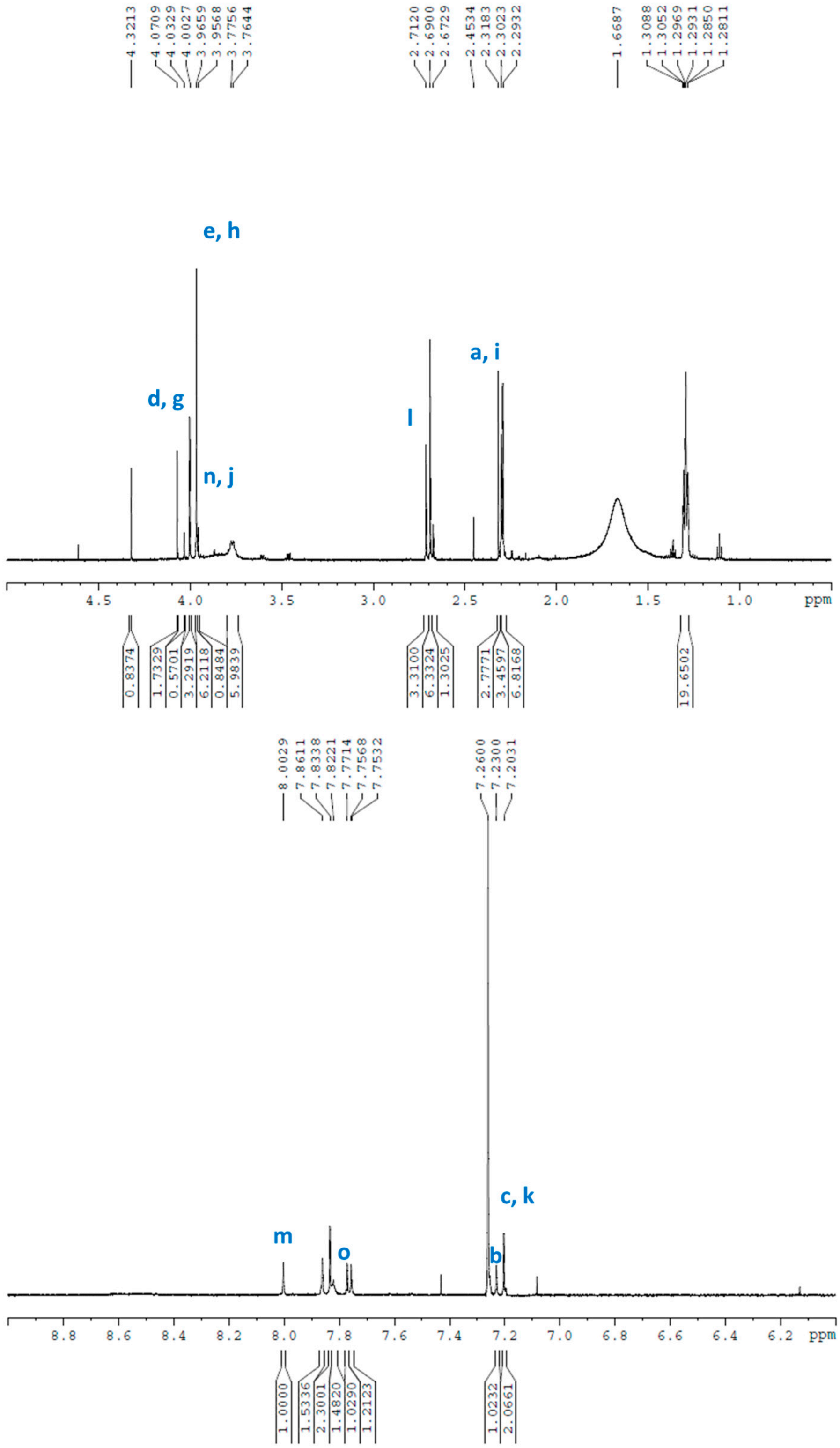


**Figure S3.** NMR analysis of dye 019-A in $CDCl_3$.

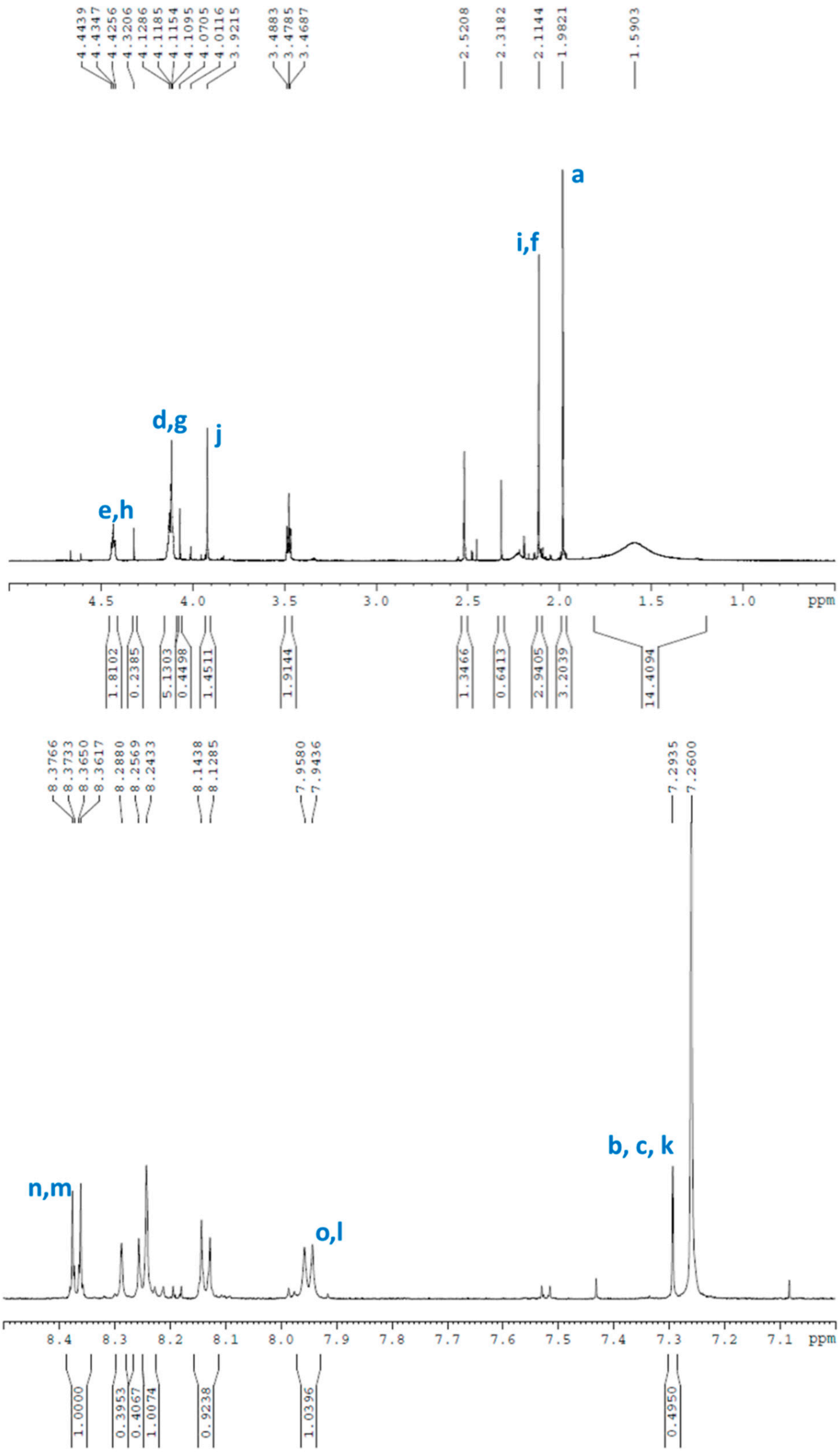


**Figure S4.** NMR analysis of dye 019-B in $CDCl_3$.

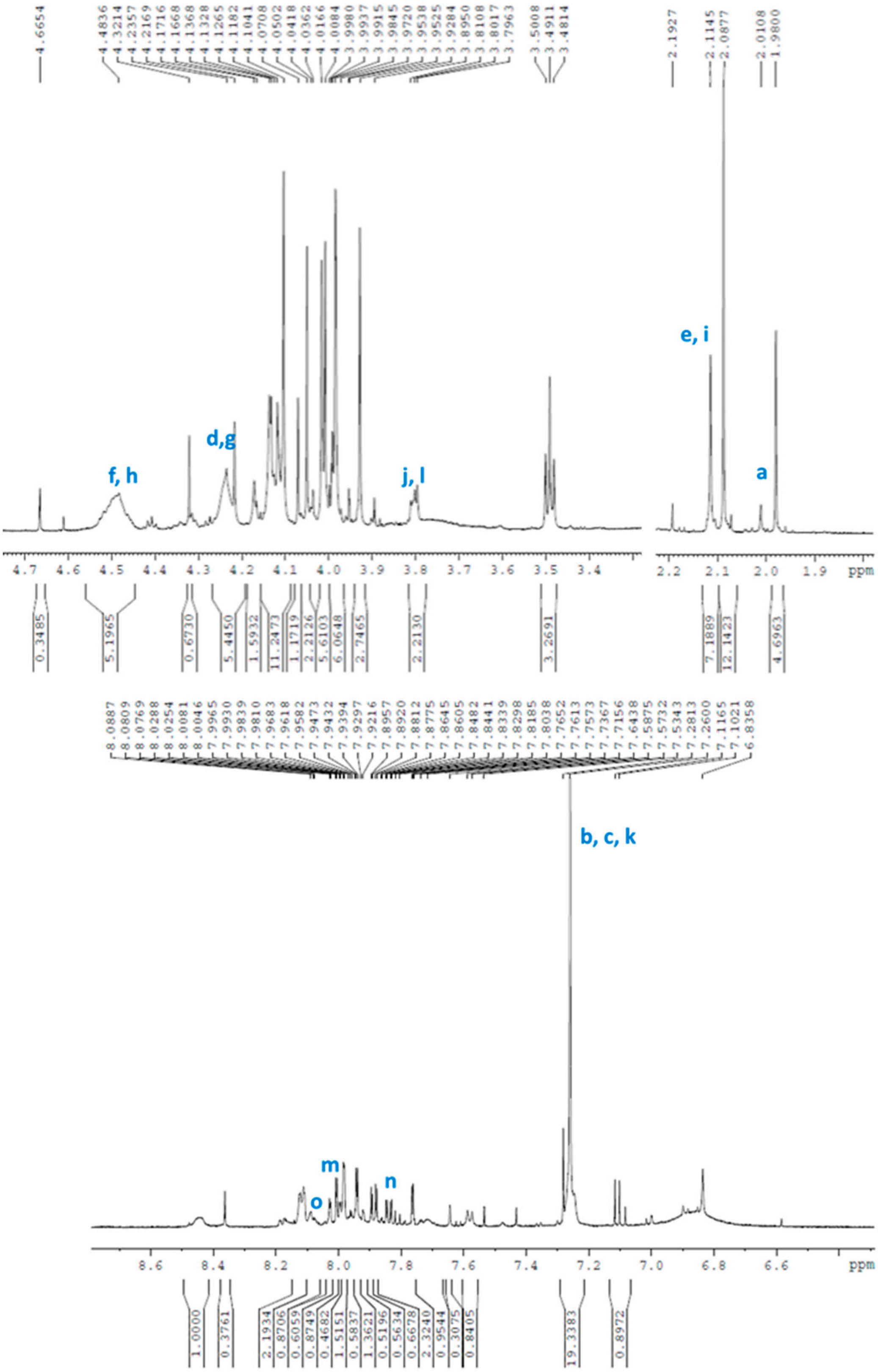


**Figure S5.** NMR analysis of dye 019-C in $CDCl_3$.

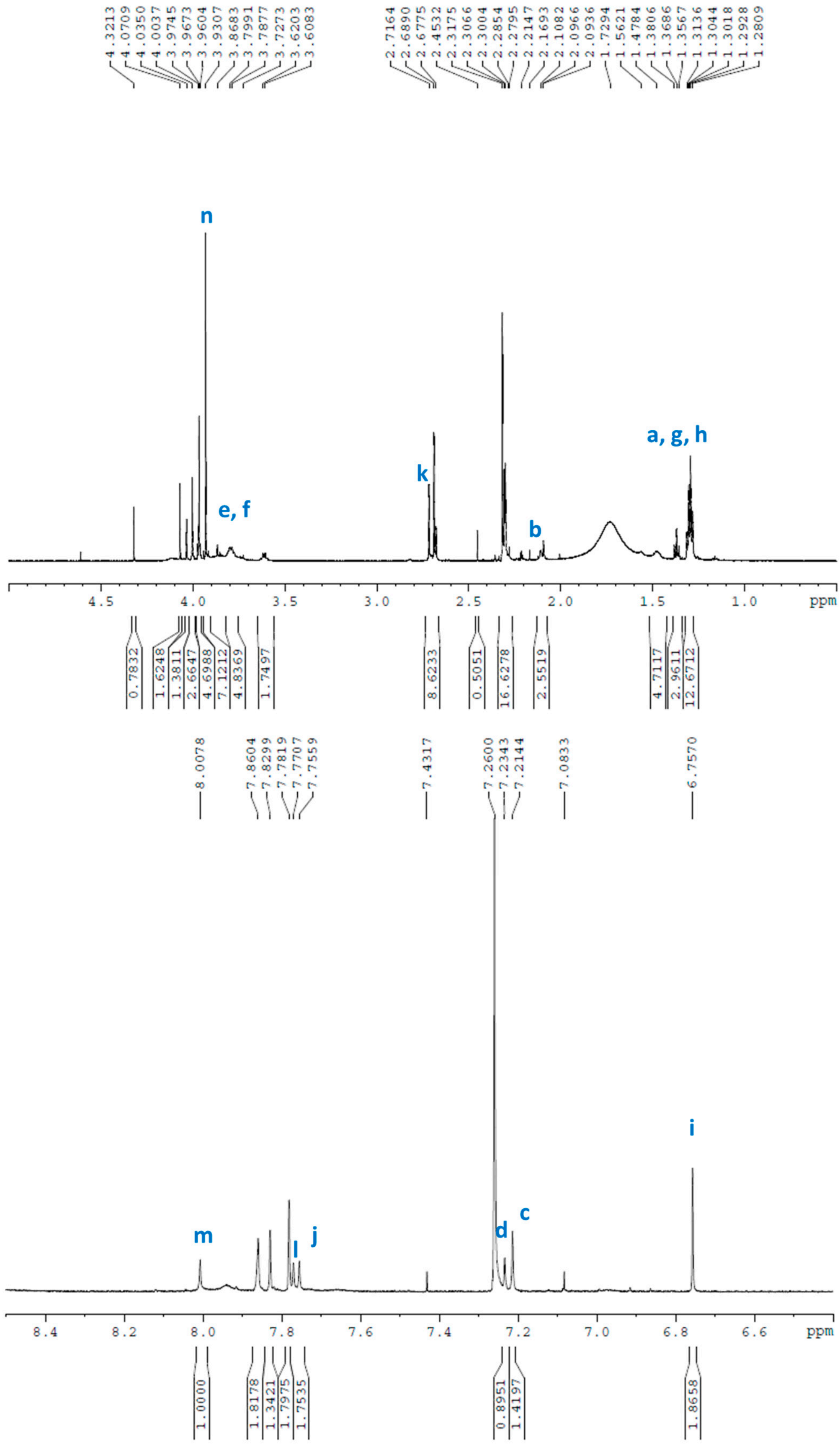


**Figure S6.** NMR analysis of dye 160-A in $CDCl_3$.

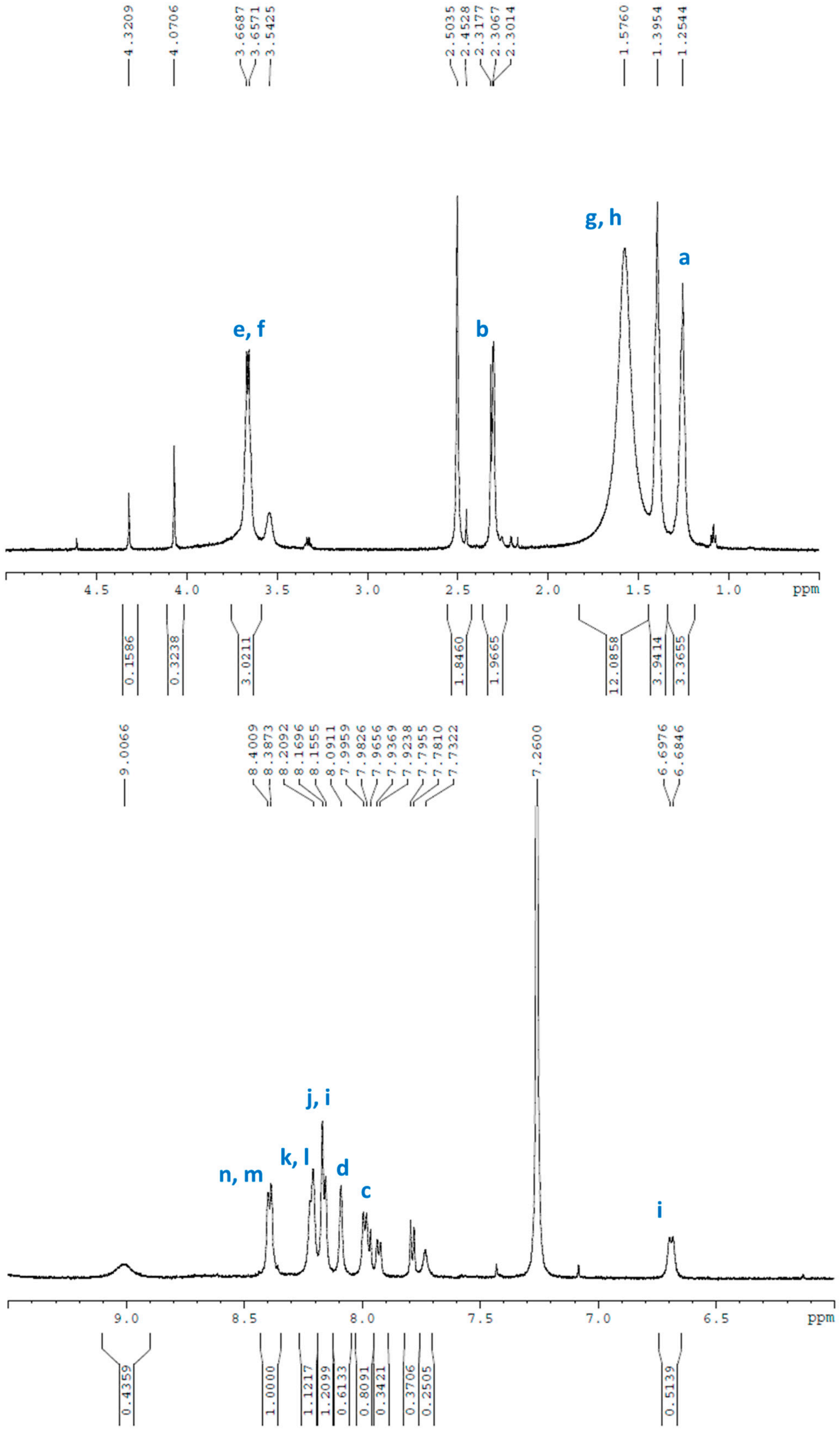


**Figure S7.** NMR analysis of dye 160-B in $CDCl_3$.

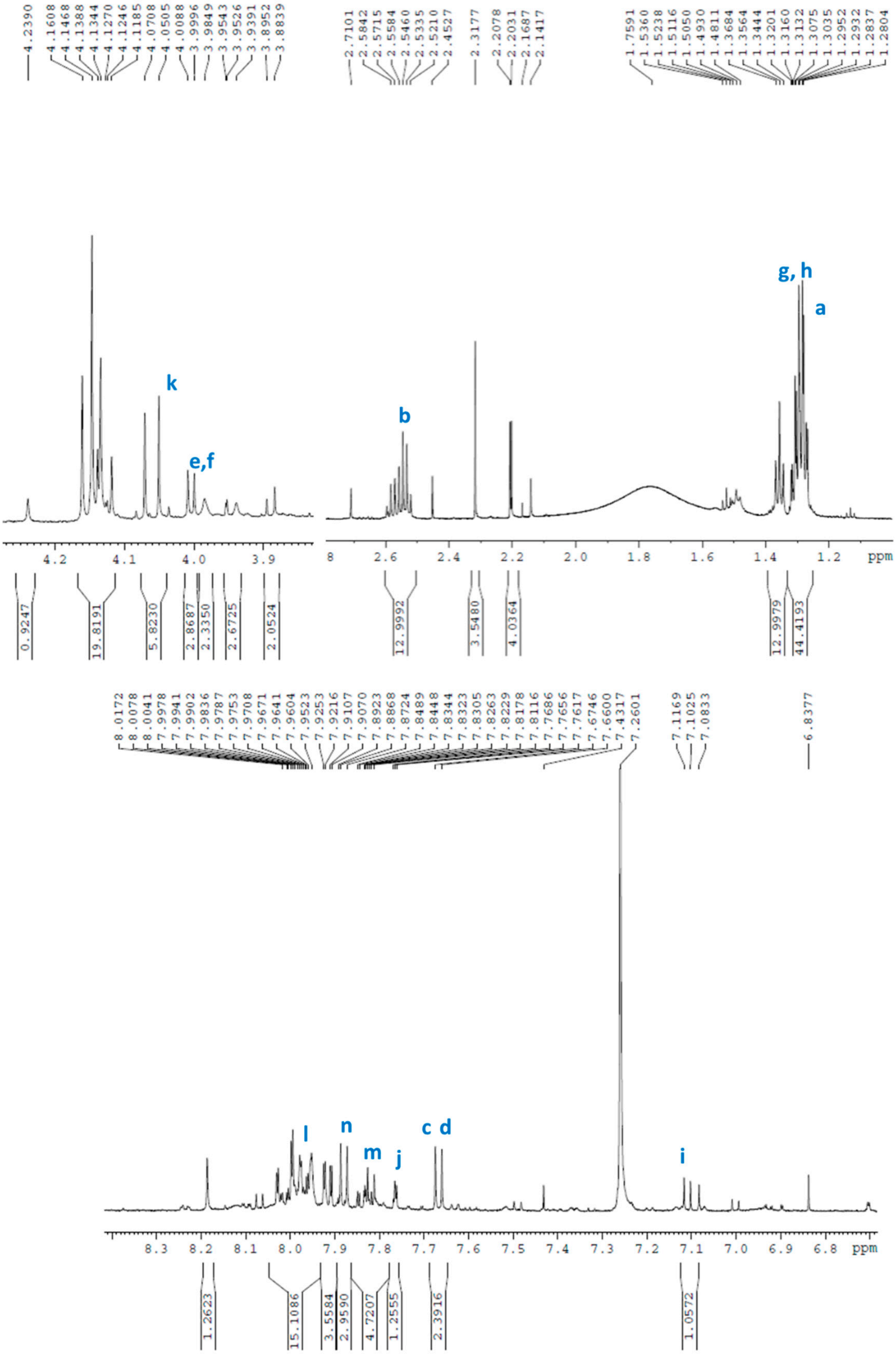


**Figure S8.** NMR analysis of dye 160-C in $CDCl_3$.

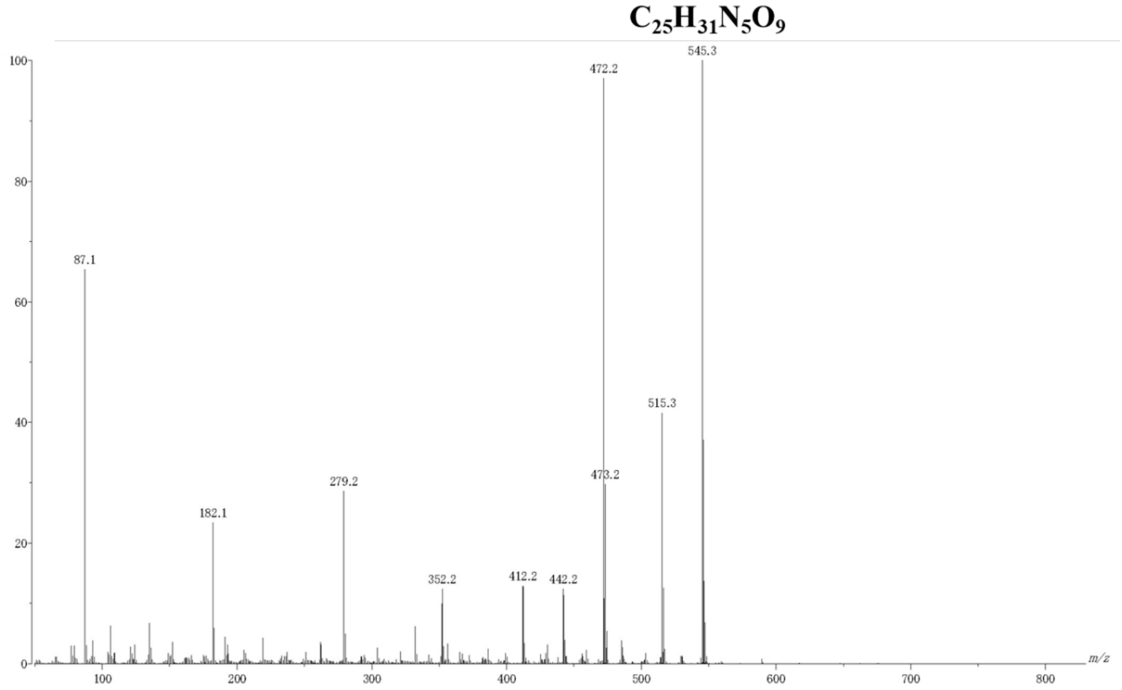


**Figure S9.** Mass spectroscopy analysis of dye 019-A.

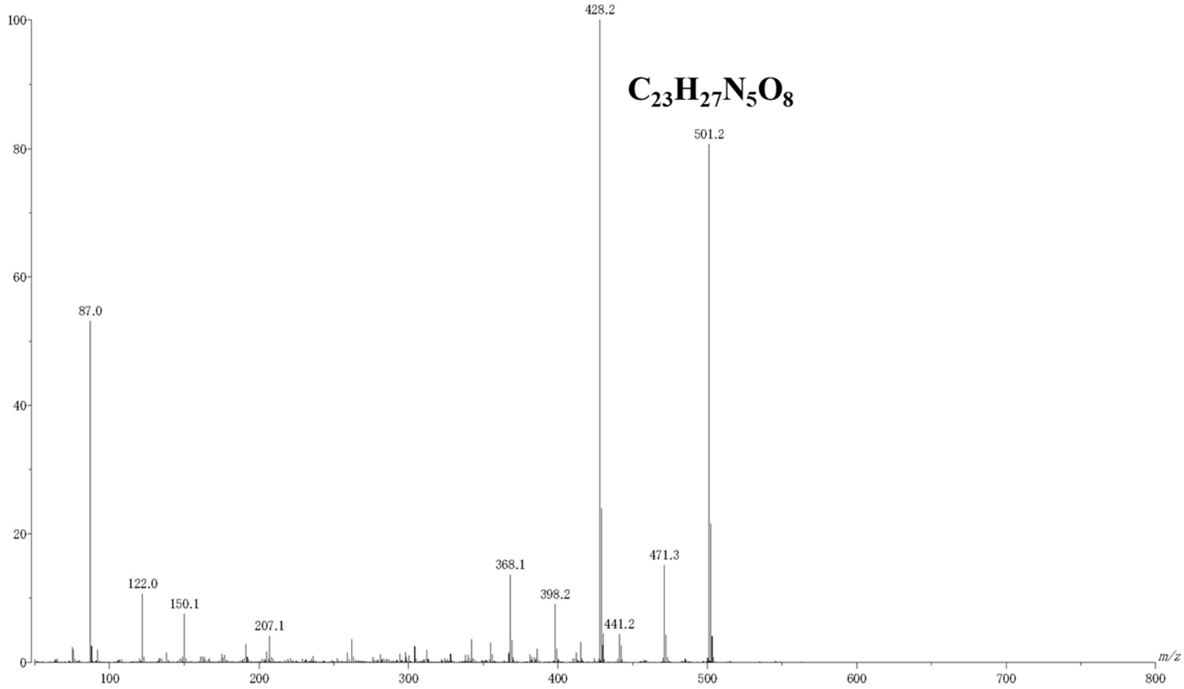


**Figure S10.** Mass spectroscopy analysis of dye 019-B.

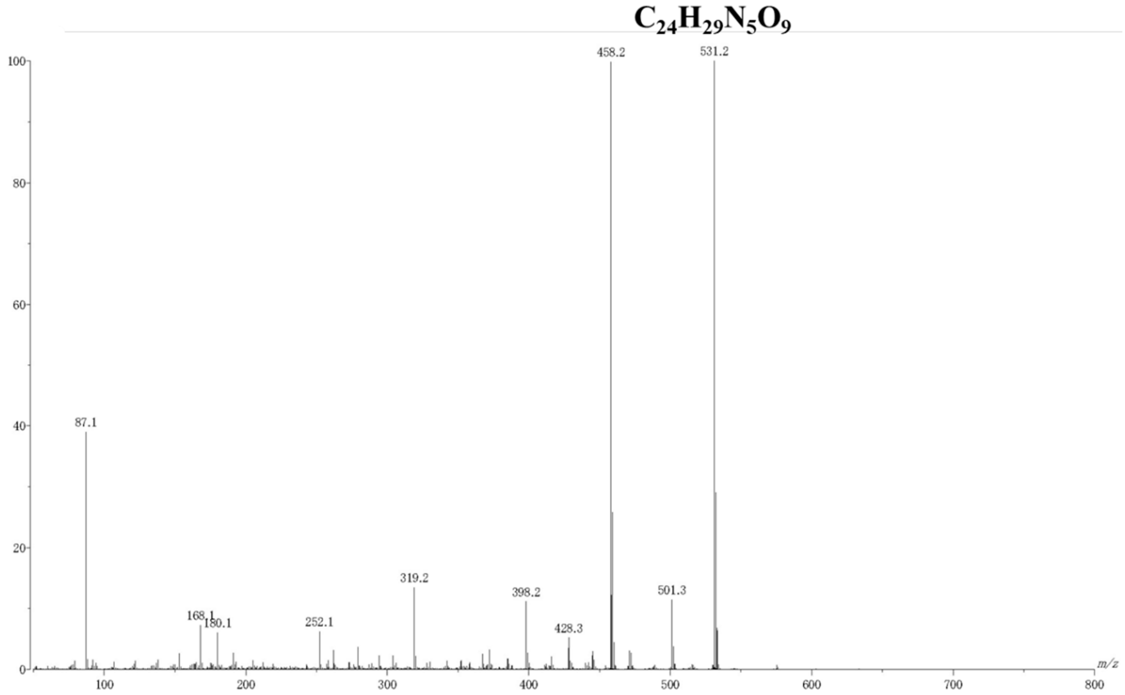


**Figure S11.** Mass spectroscopy analysis of dye 019-C.

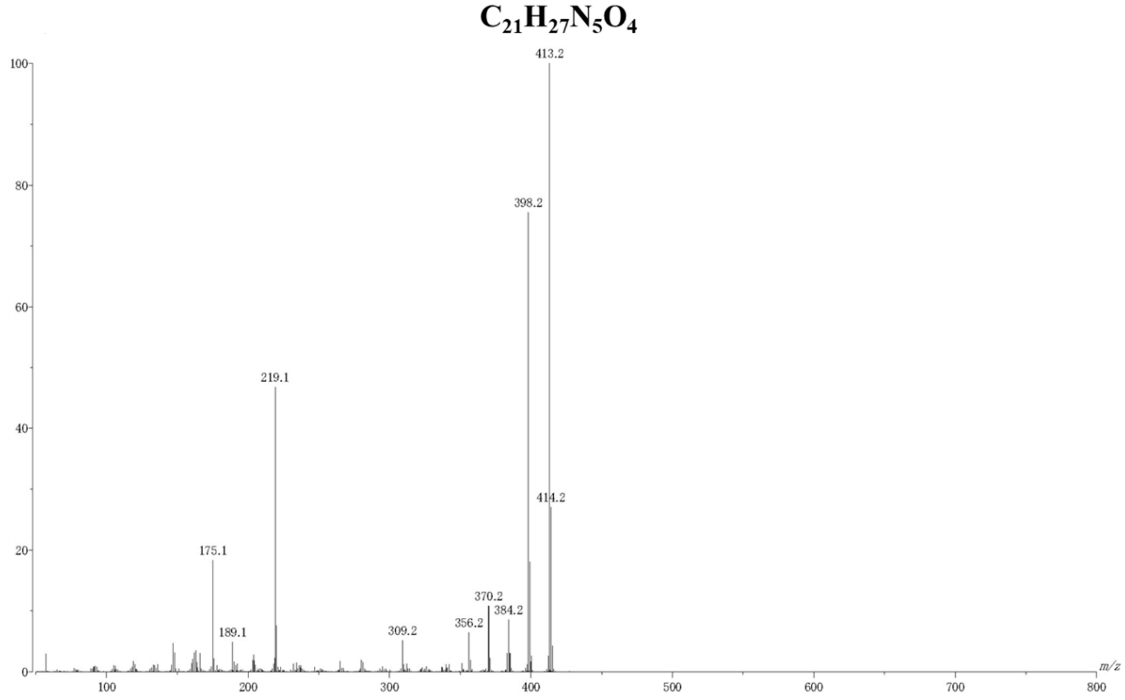


**Figure S12.** Mass spectroscopy analysis of dye 160-A.

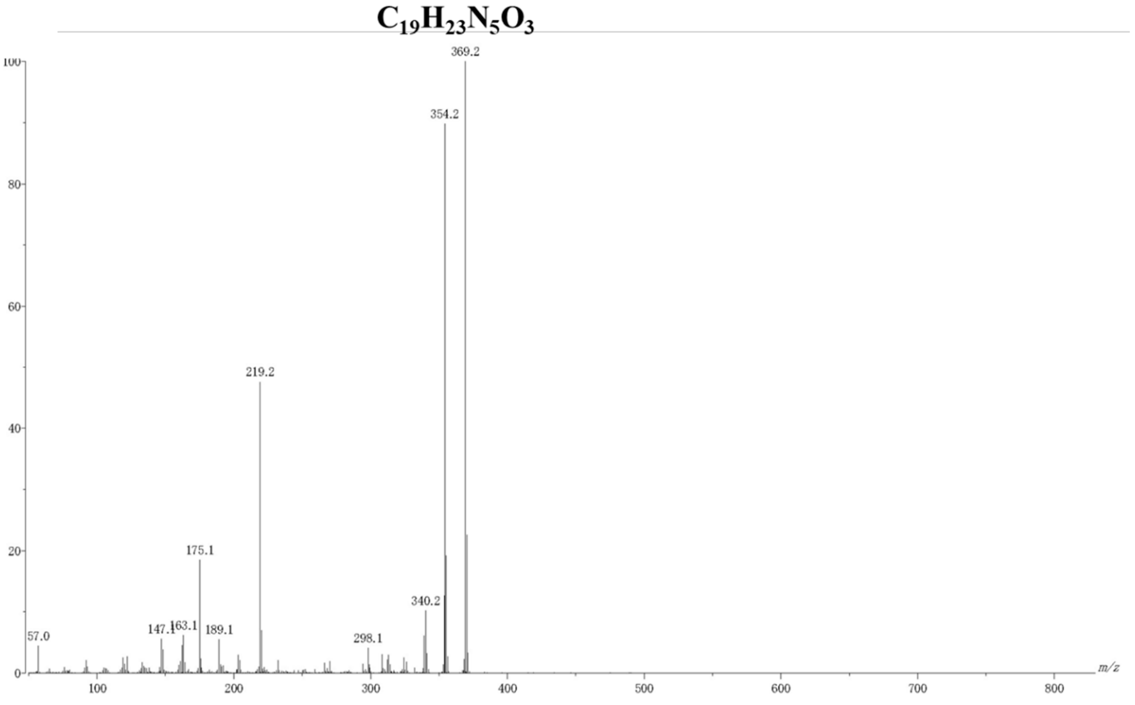


**Figure S13.** Mass spectroscopy analysis of dye 160-B.

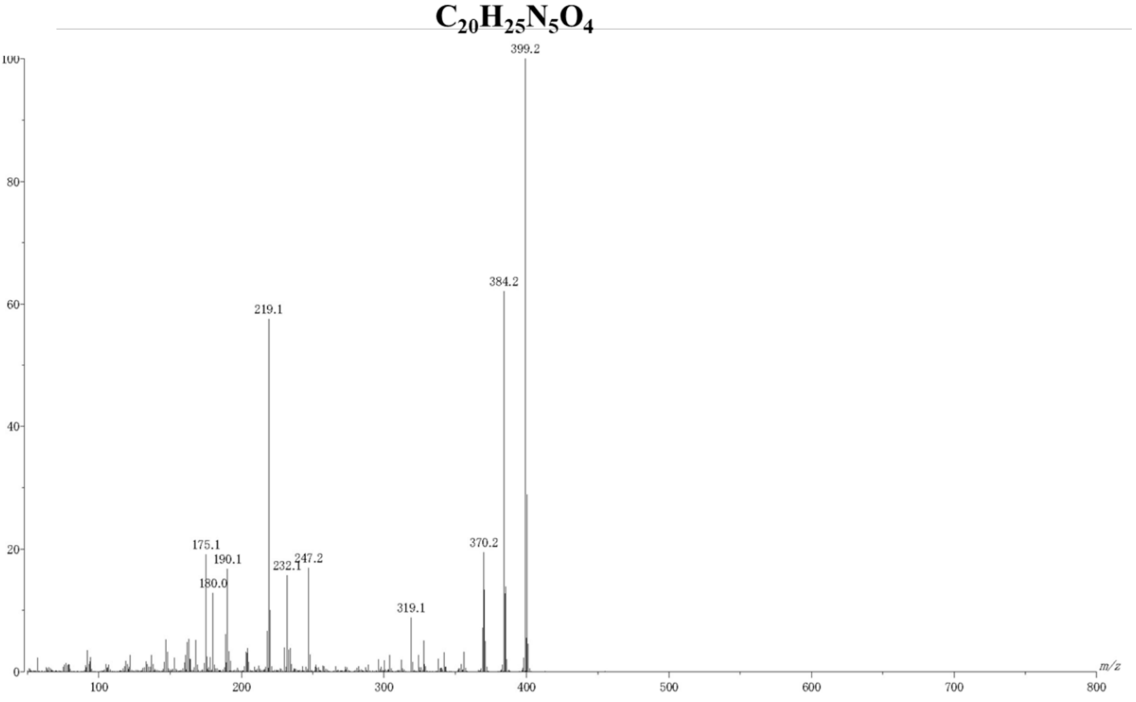


**Figure S14.** Mass spectroscopy analysis of dye 160-C.

**Table S1.** Experimental parameters of disperse dye synthesis.

| Dye | Diazo Component (g) | HCl (ml) | $H_2O$ (mL) | Sodium nitrate (g) | coupling component (g) | HAc (ml) | $H_2O$ (mL) |
|---|---|---|---|---|---|---|---|
| 019A | 0.93 | 6 | 10 | 0.75 | 1.8 | 7.5 | 2.5 |
| 019B | 1.07 | 6 | 10 | 0.74 | 1.8 | 7.5 | 2.5 |
| 019C | 1.21 | 6 | 10 | 0.77 | 1.8 | 7.5 | 2.5 |
| 160A | 1.49 | 6 | 10 | 0.74 | 1.8 | 7.5 | 2.5 |
| 160B | 1.77 | 6 | 10 | 0.78 | 1.8 | 7.5 | 2.5 |
| 160C | 2.05 | 6 | 10 | 0.80 | 1.8 | 7.5 | 2.5 |

**Table S2.** Disperse dye series NMR analysis.

| Dye | δ (ppm) |
|---|---|
| 019-A | δ2.3023(9H,s,H-a.,H-F,H-i); δ2.712(3H,s,H-l.); δ3.9568(6H,s,H-n.,H-j.); δ3.9659(6H,s,H-e.,H-h.); δ4.0027(H6,t,H-d.,H-g.); δ7.2013(2H,s,H-c,H-k); δ7.230(1H,s,H-b); δ7.7568(1H,s,H-o); δ8.0029(1H,s,H-m) |
| 019-B | δ1.9821(3H,s,H-a); δ2.1144(3H,s,H-f,H-i); δ3.9215(3H,s,H-j); δ4.1154(6H,t,H-d,H-g); δ4.4439(2H,t,H-e,H-h); δ7.2935(1H,s,H-b,H-c,H-k); δ7.9436(1H,d,H-o,H-l); δ8.3776(1H,d,H-n,H-m) |
| 019-C | δ2.0108(3H,s,H-a); δ2.1145(3H,s,H-e,H-i); δ3.8017(3H,s,H-j,H-l); δ4.2357(2H,t,H-d,H-g); δ4.4836(2H,t,H-f,H-h); δ7.2813(1H,s,H-b,H-c,H-k); δ7.8645(1H,d,H-n); δ8.0046(1H,d,H-m); δ8.0809(1H,d,H-o) |
| 160-A | δ1.3018(9H,t,H-a,H-g,H-h); δ2.1082(2H,m,H-b); δ2.7164(3H,s,H-k); δ3.7877(4H,m,H-e,H-f); δ3.9307(3H,s,H-n); δ6.7570(1H,d,H-i); δ7.2144(1H,s,H-c); δ7.2343(1H,s,H-d); δ7.7559(1H,d,H-j); δ7.7707(1H,s,H l); δ8.0078(1H,s,H-m) |
| 160-B | δ1.2544(3H,t,H-a); δ1.5760(6H,t,H-g,H-h); δ2.3177(2H,t,H-b); δ3.6571(4H,s,H-e,H-f); δ6.6976(1H,d,H-i); δ7.9959(1H,s,H-c); δ8.0911(1H,s,H-d); δ8.1696(1H,d,H-j,H-i); δ8.2092(1H,d,H-k,H-l); δ8.4009(1H,d,H-n,H-m) |
| 160-C | δ1.2952(3H,t,H-a); δ1.3684(6H,t,H-g,H-h); δ2.5584(2H,m,H-b); δ3.9996(4H,m,H-e,H-f); δ4.0505(5H,s,H-k); δ7.1169(1H,d,H-i); δ7.6600(2H,d,H-d); δ7.6746(2H,s,H-c); δ7.7656(1H,d,H-j); δ7.8489(2H,s,H m); δ7.8923(3H,d,H-n); δ7.9787(1H,d,H-l) |

**Table S3.** Mass spectrum analysis of dye 019 series.

| | | | | |
|---|---|---|---|---|
| **019A** | **$M^+$** | **M–$CH_3$** | **M–$COCH_3$** | **$C_8H_8N_3O_3$** |
| | **545.2 (50 %)** | **515.3 (13 %)** | **472.2 (100 %)** | **194.1 (6 %)** |
| 019B | $M^+$ | M–2$CH_3$ | M–$COCH_3$ | $C_6H_4N_3O_2$ |
| | 501.2 (80 %) | 471.3 (16 %) | 428.2 (100 %) | 150.1 (8 %) |
| 019C | $M^+$ | M–2$CH_3$ | M–2$CH_3$–$COCH_3$ | $C_7H_6N_3O_3$ |
| | 531.2 (50 %) | 501.3 (12 %) | 458.2 (100 %) | 180.1 (7 %) |

**Table S4.** Mass spectrum analysis of dye 160 series.

| | | | |
|---|---|---|---|
| **160A** | **$M^+$** | **M–$CH_3$** | **$C_{13}H_{17}N_2O$** |
| | **413.2 (100 %)** | **398.2 (70 %)** | **219.1 (16 %)** |
| 160B | $M^+$ | M–$CH_3$ | $C_7H_6N_3O_3$ |
| | 369.2 (80 %) | 354.2 (55%) | 150.0 (100 %) |
| 160C | $M^+$ | M–$CH_3$ | $C_7H_6N_3O_3$ |
| | 399.2 (100 %) | 384.2 (62 %) | 180.0 (13 %) |

**Table S5.** Washing fastness to detergent after dyeing according to the ISO 105 C06:2010 protocol.

| Dye | Cellulose acetate | Cotton | Nylon | Polyester | Acrylic | Wool |
|---|---|---|---|---|---|---|
| 019A | 3 | 4–5 | 3 | 4–5 | 4–5 | 4–5 |
| 019B | 3 | 4–5 | 3 | 4–5 | 4–5 | 4–5 |
| 019C | 3-4 | 4–5 | 3 | 4–5 | 4–5 | 4–5 |
| 160A | 2–3 | 4–5 | 2 | 3–4 | 4–5 | 4 |
| 160B | 2–3 | 4–5 | 2 | 4 | 4–5 | 4 |
| 160C | 3 | 4–5 | 2 | 4 | 4–5 | 4–5 |

**Table S6.** Color fastness in alkali media after dyeing according to the ISO 105 E04:2008 protocol.

| Dye | Cellulose acetate | Cotton | Nylon | Polyester | Acrylic | Wool |
|---|---|---|---|---|---|---|
| 019A | 4 | 4–5 | 4–5 | 4 | 4–5 | 5 |
| 019B | 4 | 4–5 | 3–4 | 4 | 5 | 4–5 |
| 019C | 4–5 | 4–5 | 4 | 4–5 | 5 | 5 |
| 160A | 4–5 | 5 | 4 | 4–5 | 5 | 5 |
| 160B | 4–5 | 4–5 | 4–5 | 4–5 | 5 | 4–5 |
| 160C | 4–5 | 4–5 | 4–5 | 5 | 5 | 5 |

## References

1. Zolinger, H. Color Chemistry, New York: Weinheim, VCH, chap-7, 1987.
2. Hong, Changxian, Research on the synthesis of azo dyes of aminophenol compounds and G-acid, National Central University, Institute of Chemical Engineering, Master's thesis, 2000, 4–6.
3. Huang, Bohan, Dyeing properties and functions of water-repellent dyes on PTT fibers, Master's thesis of Institute of Textile Engineering, Feng Chia University, 2004.
4. Zhang, Mingzhong, Research on Water Repellency of Fluoroalkyl Acid Dyes, Master Thesis, Institute of Textile Engineering, Feng Chia University, 1990, *1–9*, 14–18.
5. Lai, Junji, Synthesis and Application of Water- and Oil-Repellent Superfine Polyester Fiber Dyes, Master's Thesis, Institute of Organic Polymers, Taipei University of Technology, 2001.
6. Chen, Rongqi, Dyestuff Chemistry, Beijing: Textile Industry Press, 1989, 39–58.
7. He, Jinxin, Dyestuff Chemistry, Beijing: China Textile Press, 2004, 30–39.
